# Naphthalimide-Based Fluorescent Probe for Portable and Rapid Response to γ-Glutamyl Transpeptidase

**DOI:** 10.3390/molecules30153174

**Published:** 2025-07-29

**Authors:** Jinhu Wang, Xianchao Jia, Yihao Zhang, Ye Gao, Lei Zhang, Changgong Meng, Zhaohui Wang, Yang Jiao

**Affiliations:** 1 State Key Laboratory of Fine Chemical, School of Chemical Engineering, Dalian University of Technology, Dalian 116024, China; wjh937@mail.dlut.edu.cn (J.W.); xcjia@mail.dlut.edu.cn (X.J.); 1329731948@mail.dlut.edu.cn (Y.Z.); gaoye@mail.dlut.edu.cn (Y.G.); aizome@mail.dlut.edu.cn (L.Z.); cgmeng@dlut.edu.cn (C.M.); 2 Department of Gastroenterology, Central Hospital of Dalian University of Technology (Dalian Municipal Central Hospital), Dalian 116024, China

**Keywords:** fluorescent probe, GGT, cell imaging, test strip, portable detection

## Abstract

γ-Glutamyl transpeptidase (GGT) is overexpressed in a variety of diseases, making it an important diagnostic criterion for diseases. Herein, a new fluorescence probe based on naphthalimide (Glu-MDA) was developed and employed for the rapid detection of GGT in tumor cells or samples. Alkynylated naphthalimide is the fluorescent core for excellent fluorescence response. The covalent bridging of self-immolative short linkers reduces the steric hindrance between probes and enzyme cleavage sites, which leads to improved enzymatic reaction kinetics. Glu-MDA shows a rapid response and excellent selectivity with a detection limit of 0.044 U/L. This allows the efficient detection of GGT levels in solution and cells. Simultaneously, the construction of Glu-MDA pre-stained test strips provided an innovative strategy for the qualitative detection of GGT activity, helping to detect GGT faster, more portably, and cost-effectively in various scenarios.

## 1. Introduction

γ-Glutamyl transpeptidase (GGT), a protease located on the surface of the cell membrane [1], plays an important role in cell signal transduction pathways and maintaining glutathione homeostasis [2,3,4]. The presence of certain pathologies, including acute kidney injury (AKI) [5], hepatic disease [6], and cancer [7], has been observed to frequently result in elevated GGT levels. Accumulated evidence has demonstrated that the elevated levels of GGT within the tumor microenvironment significantly enhance cancer cells’ resistance to oxidative stress and toxic insults, while also facilitating their survival, invasion, and metastatic potential [8]. A strong correlation with tumor progression highlights the potential of GGT as an effective diagnostic criterion for cancer. The efficient and rapid detection of GGT levels in cells and circulation may serve as an early warning mechanism, allowing timely intervention and improving prognosis [9,10]. Nevertheless, the current detection methods of GGT, including colorimetric assays [11], the enzyme-linked immunosorbent assay (ELISA) [12], and electrochemical methods [13], are limited by insufficient sensitivity, cumbersome operation steps, and a dependence on specialized equipment, which lead to low diagnostic efficiency. Consequently, the development of a low-cost, portable, and rapid assay method for GGT activity in cells or samples is imperative for disease diagnosis.

Fluorescent imaging technology, distinguished by its good sensitivity, non-destructiveness, and rapid detection capabilities, has attracted much attention for the detection of disease-related enzymes [14]. Fluorescent probes, the core component of fluorescence imaging technology, are primarily composed of fluorophores, linkers, and recognition groups [15,16]. Based on the catalytic properties of GGT, the γ-glutamyl group can serve as a recognition group in a GGT-responsive fluorescent probe, as GGT can efficiently cleave the amide bond within the γ-glutamyl group when the γ-glutamyl group enters the active pocket of GGT [17]. Additionally, the rational design and introduction of linkers in the probe can effectively reduce steric hindrance, consequently improving the reaction rate [18]. Notably, 4-aminobenzyl alcohol holds potential as a self-immolative linker due to its unique electron transfer properties [19]. Fluorophores such as coumarin, rhodamine, naphthalimide, cyanine, and BODIPY serve as core building blocks for fluorescent probes due to their fluorescence properties, with naphthalimide emerging as an ideal candidate for fluorophores, owing to its exceptional light stability and ease of modification [20,21]. Consequently, the rational integration of functional groups into a naphthalimide-based fluorescent probe is anticipated to enable the rapid detection of GGT and facilitate disease diagnosis efficiency.

**Scheme 1:**
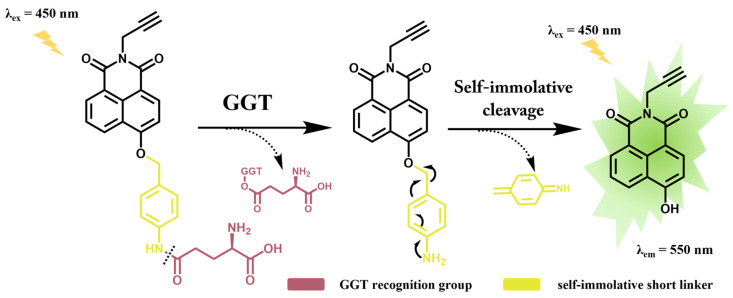
Proposed sensing mechanism of Glu-MDA towards GGT.

Herein, a naphthalimide-based GGT-responsive fluorescent probe, Glu-MDA, was proposed for the purpose of detecting GGT levels in cells and liquid samples. Glu-MDA utilizes 4-hydroxynaphthalimide, a compound with sufficient photostability, which ensures efficient and stable imaging performance in aqueous biological environments. The covalent bridging of the hydroxyl auxochrome and a self-immolative short linker mitigates the steric hindrance caused by the rigid conjugated plane, which ensures the smooth progression of GGT enzymatic cleavage and activation fluorescence processes. Upon Glu-MDA entering the active pocket of GGT, the amide bond of Glu-MDA is cleaved by the active site of GGT, thereby initiating a cascade reaction and releasing the fluorophore, thus achieving fluorescence recovery (Scheme 1). Glu-MDA can detect GGT over a wide concentration range, with good selectivity and low detection limits. Glu-MDA can effectively monitor GGT activity in physiological environments. Furthermore, a fluorescent response test strip for the portable and rapid detection of GGT levels was developed by utilizing the excellent color development ability of Glu-MDA toward GGT, which can serve as a reliable auxiliary index for disease diagnosis.

## 2. Results and Discussion

**Scheme 2:**
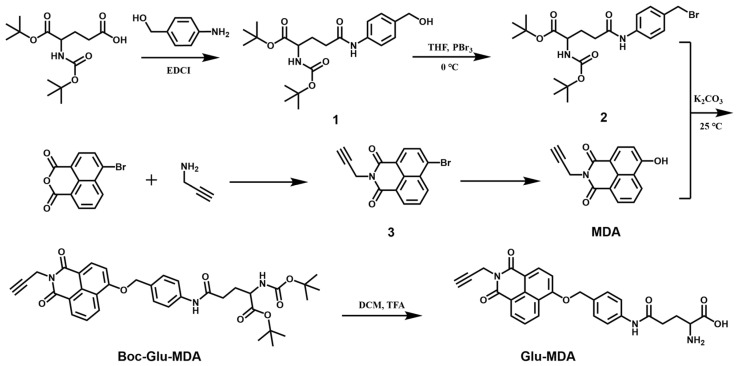
Synthetic route of probe Glu-MDA.

The fluorescent probe Glu-MDA was prepared by a six-step synthesis reaction, and the synthetic route is shown in Scheme 2. ^1^H NMR, ^13^C NMR, and HRMS of the compounds are provided in the Supplementary Materials.

### 2.1. Optical Characteristics of Fluorophore MDA and Probe Glu-MDA

**Figure 1:**
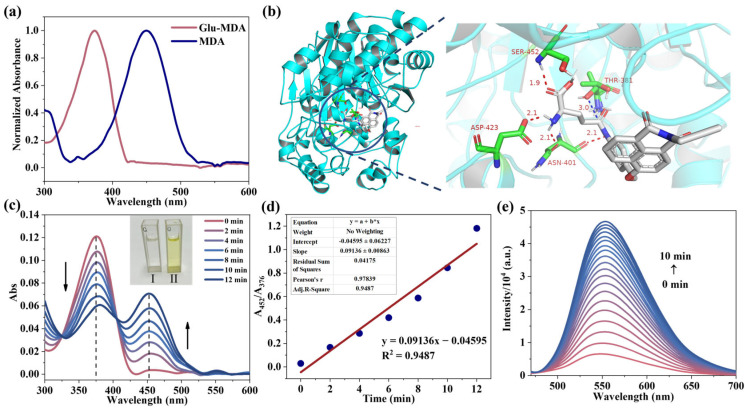
(**a**) The normalized absorption spectra of probe Glu-MDA (10 μM) and fluorophore MDA (10 μM) in DMSO/PBS (1:4 *v*/*v*) mixed solvent. (**b**) Molecular docking results of probe Glu-MDA and GGT. (**c**) The UV absorption of the probe Glu-MDA (10 μM) changes over time after the addition of GGT (2 U/L). Illustration: solution image before and after adding GGT. I: before adding GGT; II: after adding GGT. (**d**) The linear relationship between A_452_/A_376_ and time after adding GGT (2 U/L) to the probe Glu-MDA (10 μM) solution. (**e**) The fluorescence spectra of the probe Glu-MDA (10 μM) changes over time in DMSO/PBS (1:4 *v*/*v*) buffer after the addition of GGT (2 U/L); λ_ex_ = 450 nm.

Initially, the absorbance, normalized excitation, and emission spectra of the fluorescent group MDA and the probe Glu-MDA were tested and compared to confirm the changes in the photophysical properties after the modification of the fluorescent groups. The UV absorption capacity of the fluorophore MDA (10 μM) and the probe Glu-MDA (10 μM) in various solvents was analyzed. As shown in Figure S15, both MDA and Glu-MDA demonstrate notable UV absorption capacity in various solvents. To detect GGT in a cellular environment, a biological environment was simulated to investigate the UV absorption changes in the probe Glu-MDA (10 μM) and the fluorophore MDA (10 μM) in a DMSO/PBS (1:4 *v*/*v*) mixed solvent [22]. A pronounced blue shift was observed in the maximum absorption wavelength (λ_max_) of the probe Glu-MDA, shifting from 452 nm to 376 nm compared to the fluorophore MDA (Figure 1a). The maximum excitation wavelengths for the fluorophore MDA and the probe Glu-MDA were determined to be 370 nm and 450 nm, respectively. A significant blue shift in the maximum excitation wavelength of Glu-MDA was observed (Figure S16). Exciting Glu-MDA and MDA at 450 nm resulted in MDA exhibiting significantly higher fluorescence intensity than Glu-MDA (Figure S17). The observed difference in optical properties suggests that Glu-MDA has potential as a fluorescent probe.

Stable fluorescence detection requires fluorophores with good stability to support the retention of the detection results. The fluorophore MDA demonstrated no significant alteration in its maximum absorption wavelength in a mixed solvent of DMSO/PBS (1:4 *v*/*v*) over the course of time, thereby suggesting that the fluorophore MDA possessed stable photophysical properties (Figure S18). The fluorophore MDA exhibited a negligible reduction in fluorescence intensity over a 2 h period (Figure S19), thereby demonstrating its potential for GGT detection using the probe Glu-MDA. Given that tumors possess a mildly acidic internal microenvironment [23,24], the photostability of MDA was systematically evaluated across different pH levels. As shown in Figure S20, the results reveal that within a broad pH range (5–9), the fluorescence intensity of MDA remains largely unchanged, thereby confirming its fluorescence stability within the acidic tumor microenvironment.

### 2.2. Probe Glu-MDA and GGT Simulated Molecular Docking

The effective binding of probes to GGT recognition sites is the key to activating fluorescence for detection. Here, Autodock 4.2 is utilized to simulate and predict binding patterns between proteins and small molecules [25,26] in order to evaluate the feasibility of Glu-MDA as a probe for identifying and detecting GGT. In the selection of the crystal structure for GGT, the glutamic acid binding site was taken into consideration. The crystal protein structure (PDB: 4GDX) of GGT in complex with glutamic acid was selected to facilitate the analysis of the docking results. The crystal structure of GGT served as the receptor, while Glu-MDA acted as the ligand in semi-flexible docking simulations. By refining and visualizing the GGT crystal model, observing the docking pocket of the protein crystal, and establishing the docking box at the docking pocket, Glu-MDA underwent 200 independent docking experiments. The most stable binding configuration was determined by comparing the docking results with glutamic acid. A subsequent analysis revealed that the binding energy between Glu-MDA and GGT active site residues was −9.43 kcal/mol. As visualized in Figure 1b, the molecular docking results indicated that the amino hydrogen atom of the Glu-MDA recognition group engaged in hydrogen bonding with the carboxyl oxygen atom of the ASP-423 residue and the acyl oxygen atom of the ASN-401 residue. The amino hydrogen atom at the cleavage site was found to be involved in a hydrogen bond with another oxygen atom of the residue ASN-401. The hydrogen bond distances described above are all approximately 2.1 Å. Meanwhile, the oxygen atom of the carboxyl group in the recognition group formed a stable hydrogen bond with the amino hydrogen atom of SER-452. The distance of this hydrogen bond is approximately 1.9 Å. (red dotted line) [27,28]. The probe Glu-MDA was shown to form stable hydrogen bonds with the GGT active pocket, thereby validating its potential as an effective probe for GGT. Furthermore, the spatial distance between the GGT active site (THR-381) and the Glu-MDA amide bond is a pivotal factor in the enzymatic reaction. Specifically, the distance between the oxygen atom in the THR-381 side chain and the amide bond carbon atom in the Glu-MDA recognition group is approximately 3 Å (blue dotted line), indicating the potential for the formation of an acyl bond between the two, which is a prerequisite for GGT to catalyze Glu-MDA [29]. The aforementioned results provide a solid theoretical foundation for subsequent experimental research.

### 2.3. Spectral Response of Probe Glu-MDA to GGT

**Figure 2:**
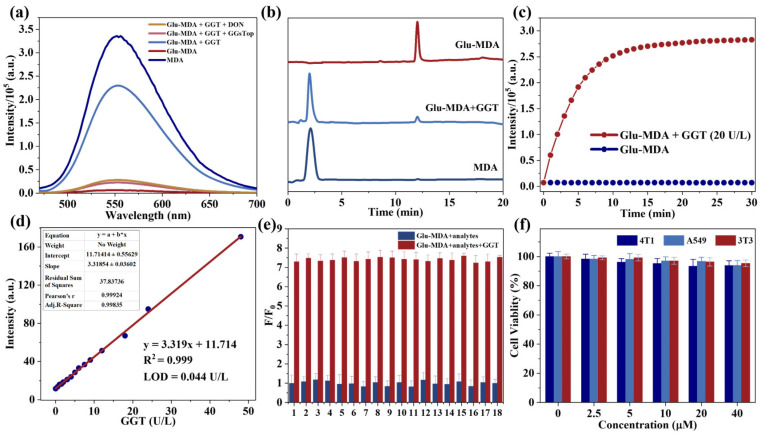
(**a**) The fluorescence spectra of MDA and Glu-MDA under different treatments: MDA (10 μM), Glu-MDA (10 μM), Glu-MDA (10 μM) + GGT (10 U/L), Glu-MDA (10 μM) + GGT (10 U/L) + GGsTop (0.1 mM), and Glu-MDA (10 μM) + GGT (10 U/L) + DON (0.1 mM). λ_ex_ = 450 nm and λ_em_ = 550 nm. (**b**) HPCL analysis of Glu-MDA, Glu-MDA + GGT, and MDA. (**c**) Fluorescence kinetics of Glu-MDA (10 µM) on GGT (20 U/L). λ_ex_ = 450 nm and λ_em_ = 550 nm. (**d**) Glu-MDA probe detection limit for GGT. (**e**) Fluorescence response of Glu-MDA (10 μM) to GGT (2 U/L) and various analytes at 550 nm. Analytes 1–18: None, NaNO_3_, Na_2_SO_3_, NaCl, KCl, CaCl_2_, H_2_O_2_, L-ascorbic acid, Glucose, Phenylalanine (Phe), Proline (Pro), Alanine (Ala), Homocysteine (Hcy), Glutathione (GSH), Glutathione Reductase (GR), Glutathione Peroxidase (GSH-Px), Glutathione S-transferase (GST), and Glutamate Cysteine Ligase (GCL). Data are presented as the mean value ± SD (n = 3). (**f**) Cytotoxicity of different concentrations of Glu-MDA on 4T1 cells, A549 cells, and 3T3 cells. Data are presented as mean ± standard deviation (SD) (n = 3).

With the support of simulated theoretical data, the response of Glu-MDA (10 μM) to GGT (2 U/L) in a mixed solution was investigated using ultraviolet absorption spectroscopy and fluorescence spectroscopy. Upon the addition of GGT, the absorption peak at 376 nm, which corresponds to Glu-MDA, gradually weakened over time. Simultaneously, a new peak emerged at 452 nm, attributable to the absorption of fluorophores generated during the reaction (Figure 1c). A further comparison of the absorption intensities at 376 nm and 452 nm revealed a linear relationship with the reaction time (Figure 1d and Figure S21). Moreover, the solution’s color transitioned from colorless to pale yellow (Figure 1c). Subsequently, a fluorescence analyzer was used to measure the fluorescence response of the probe Glu-MDA (10 μM) upon the addition of GGT (2 U/L). The results demonstrated that the fluorescence intensity at 550 nm increased gradually (Figure 1e). Furthermore, the fluorescence spectrum of the probe Glu-MDA after reacting with GGT exhibited a peak shape and emission position that closely matched those of the fluorophore’s fluorescence spectrum (Figure 2a). It is noteworthy that the probe Glu-MDA exhibited no recovery of fluorescence in the presence of GGT inhibitors, such as GGsTop and 6-diazepine-5-oxo-L-norleucine (DON) (Figure 2a). The aforementioned results indicate that Glu-MDA exhibits a specific response to GGT.

### 2.4. Reaction Mechanism

A more profound comprehension of the enzyme activation mechanism of the probe Glu-MDA, utilized for monitoring GGT activity, is of paramount importance for a more profound elucidation of the probe’s operational principle. To this end, we conducted high-performance liquid chromatography (HPLC) characterization experiments [30,31]. As shown in Figure 2b, the probe Glu-MDA displayed an endogenous signal peak with a retention time of approximately 12.0 min. After the addition of GGT and subsequent incubation, a new signal peak emerged at approximately 2.1 min, showing good agreement with the retention time of the fluorophore. Simultaneously, the original peak intensity decreased significantly. These results indicate that when the probe Glu-MDA interacts with GGT, the fluorophore MDA is released.

### 2.5. Kinetics

Subsequently, the fluorescence intensity change rate of the probe at 550 nm was explored under the catalysis of different enzyme concentrations. The results obtained demonstrated a direct correlation between the reaction rate and enzyme concentration, thereby confirming the enzyme concentration dependence of the reaction (Figure S22). Concurrently, the fluorescence intensity of the probe Glu-MDA (10 μM) exhibited a gradual stabilization within 15 min following the addition of varying concentrations of GGT (Figure 2c and Figure S22). This outcome can be attributed to the incorporation of 4-aminobenzyl alcohol, which effectively reduces the steric hindrance of the probe in relation to the enzyme [32]. As a result, these results indicate that Glu-MDA demonstrates a significant and rapid response to GGT, thus highlighting its promising potential for applications in biological systems.

### 2.6. Limit of Detection

In the subsequent experiment, the detection limit of the probe Glu-MDA for GGT was measured. To this end, different concentration groups of GGT were set, and the probe Glu-MDA (10 μM) was incubated with the samples for 5 min. The fluorescence signal was then recorded using a microplate reader. The signal quantification revealed a robust linear correlation between fluorescence intensity and GGT concentration (0–48 U/L), with a calculated detection limit (LOD) of 0.044 U/L (Figure 2d), signifying a lower detection limit compared to those of previously documented GGT-responsive fluorescent probes [33,34,35]. This result indicates that the probe Glu-MDA is a highly sensitive fluorescent probe for the detection of GGT.

### 2.7. Selectivity and Anti-Interference

In consideration of the intricacies inherent in the detection environment, a range of prevalent intracellular analytes and other enzymes related to glutathione metabolism were examined to investigate the selectivity of the probe Glu-MDA [36,37]. In the presence of various analytes, Glu-MDA was added to the solvents, and subsequent alterations in fluorescence were monitored through the utilization of a fluorescence analyzer. The results are displayed in Figure 2e and Figure S23a. The fluorescence intensity of the probe Glu-MDA exhibited minor changes in many other analytes, but in the presence of GGT was a significant enhancement in fluorescence intensity observed. This finding indicates that GGT possesses good selectivity for the probe Glu-MDA. Subsequently, the detection of GGT (2 U/L) by the probe Glu-MDA in the presence of the above analytes was investigated to evaluate potential interference [38]. The results demonstrated that the probe Glu-MDA exhibited a significant fluorescence response to GGT in the presence of a competitor, as evidenced by the comparison with the fluorescence intensity observed in the absence of the analyte (Figure 2e and Figure S23b). This finding confirmed the specific reaction between Glu-MDA and GGT and provided evidence for the potential application of the probe in biological environments.

### 2.8. Cytotoxicity

Prior to the cell imaging experiment, the physiological toxicity of the probe Glu-MDA was systematically evaluated using the methylthiazolyldiphenyl-tetrazolium bromide (MTT) assay [39]. Specifically, 3T3 cells (mouse fibroblasts), A549 cells (human lung cancer cells), and 4T1 cells (mouse breast cancer cells) were incubated with Glu-MDA at concentrations ranging from 0 μM to 40 μM (0, 2.5, 5, 10, 20, and 40 μM) for 24 h. Subsequently, their viability was quantitatively assessed via the MTT method. The results revealed that even at higher concentrations, both normal and tumor cells maintained survival rates exceeding 90%, indicating that the probe Glu-MDA exhibits negligible cytotoxicity under the experimental conditions used for cell imaging (Figure 2f and Figure S24). This evidence supports the promising biological application potential of the probe Glu-MDA.

### 2.9. Fluorescence Imaging in Living Cells

**Figure 3:**
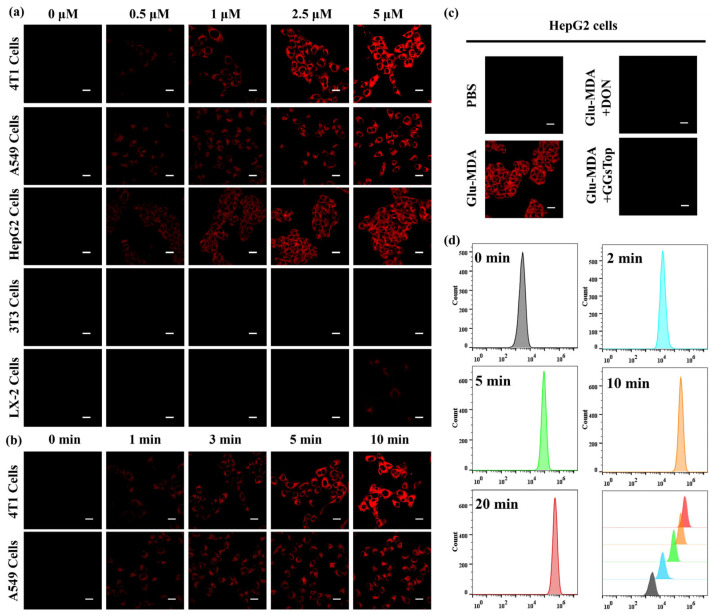
(**a**) Fluorescence imaging of endogenous GGT in 4T1 cells, A549 cells, HepG2 cells, 3T3 cells, and LX-2 cells labeled with Glu-MDA at different concentrations. λ_ex_ = 458 nm, red channel (490–600 nm). Scale bar = 20 μm. (**b**) Fluorescence imaging of 4T1 cells and A549 cells after incubation with Glu-MDA (2.5 μM) for different times, λ_ex_ = 458 nm, red channel (490–600 nm). Scale bar = 20 μm. (**c**) Fluorescence image of Glu-MDA on HepG2 cells in the presence of GGT inhibitor (1 mM), λ_ex_ = 458 nm, red channel (490–600 nm). Scale bar = 20 μm. (**d**) Flow cytometry analysis of GGT labeling in live A549 cells incubated with the probe Glu-MDA (5 μM) for different periods of time; λ_ex_ = 488 nm and λ_em_ = 533 nm.

Building upon the findings of the preceding experiments, we proceeded to utilize Glu-MDA for the imaging of both normal and tumor cells. Initially, the imaging effect of different concentrations of Glu-MDA on GGT in cells was explored. LX-2 cells (human hepatic stellate cells), 3T3 cells, A549 cells, 4T1 cells, and HepG2 cells (human hepatocellular carcinoma cells) were exposed to 0, 0.5, 1, 2.5, and 5 μM of Glu-MDA for a duration of 5 min. Thereafter, the intracellular fluorescence response was observed by laser confocal microscopy. The results demonstrated that 3T3 cells exhibited no discernible fluorescence response upon treatment with Glu-MDA (Figure 3a and Figure S25), while LX-2 cells showed a weak fluorescence signal with the increase in Glu-MDA concentration (Figure 3a and Figure S26). In contrast, A549 cells, 4T1 cells, and HepG2 cells demonstrated a pronounced fluorescence response, with the most significant response observed following incubation with 5 μM of Glu-MDA (Figure 3a and Figures S27–S29). This finding is consistent with the observed trend in previous reports. In comparison with tumor cells, GGT expression is lower in normal cells [40]. This finding confirms that the probe Glu-MDA is capable of accurately identifying GGT and effectively distinguishing tumor cells from normal cells.

To further investigate the effect of Glu-MDA incubation time on cell imaging, A549 cells and 4T1 cells were divided into five groups and incubated with 2.5 μM of Glu-MDA for 0, 1, 3, 5, and 10 min, respectively. Subsequently, cell imaging was performed using fluorescence microscopy. The results demonstrated that over time, the fluorescence in Glu-MDA-treated A549 cells and 4T1 cells progressively increased, and the cells exhibited an evident imaging effect within 3 min of incubation (Figure 3b, Figures S30 and S31). This finding suggests that the probe Glu-MDA has a rapid response ability in cell imaging.

In order to exclude potential interference from other enzymes in tumor cells on the fluorescence response of Glu-MDA, HepG2 cells were selected as a model system. The cells were pretreated with DON and GGsTop. These inhibitors were used to evaluate the fluorescence response characteristics of Glu-MDA under conditions of GGT inhibition. The experimental results revealed that the fluorescence intensity of cells treated with DON and GGsTop was significantly lower than that of untreated cells (Figure 3c). This observation confirms that Glu-MDA only responds to GGT.

### 2.10. Flow Cytometry

Concurrently, the potential of Glu-MDA for GGT detection was further evaluated by flow cytometry [41]. A549 cells were exposed to 5 μM of Glu-MDA for varying durations. Afterward, the cells were collected, and the number of stained cells were recorded. The results demonstrated that, in comparison with the blank group, the fluorescence intensity and the number of stained cells increased significantly over time, thereby validating the feasibility of Glu-MDA for detecting GGT in tumor cells by flow cytometry (Figure 3d).

### 2.11. Cell Imaging Under Sodium Butyrate Stimulation

To further examine the applicability of the probe Glu-MDA for detecting endogenous GGT activity, we assessed the intracellular GGT levels induced by sodium butyrate (NaBu). NaBu exerts its function by selectively inhibiting histone deacetylases (HDACs), enzymes that play a critical role in the opening of nucleosome structures, thereby facilitating the transcription of numerous genes, including GGT [42]. To achieve this objective, HepG2 cells were pretreated with varying incubation times in a cell culture medium containing 2.5 mM NaBu, followed by incubation with 5 μM of the probe Glu-MDA at 37 °C for 10 min. The detection capability of the probe Glu-MDA for GGT activity was subsequently utilized to monitor changes in GGT expression in cells exposed to NaBu for different durations. As shown in Figures S32 and S33, the fluorescence signal increased with prolonged exposure to NaBu, compared to the blank control group. These results clearly demonstrate that the probe Glu-MDA is capable of detecting changes in endogenous GGT activity stimulated by NaBu, thereby validating its applicability for such measurements.

### 2.12. Retention Ability of Fluorescent Reaction Products

**Figure 4:**
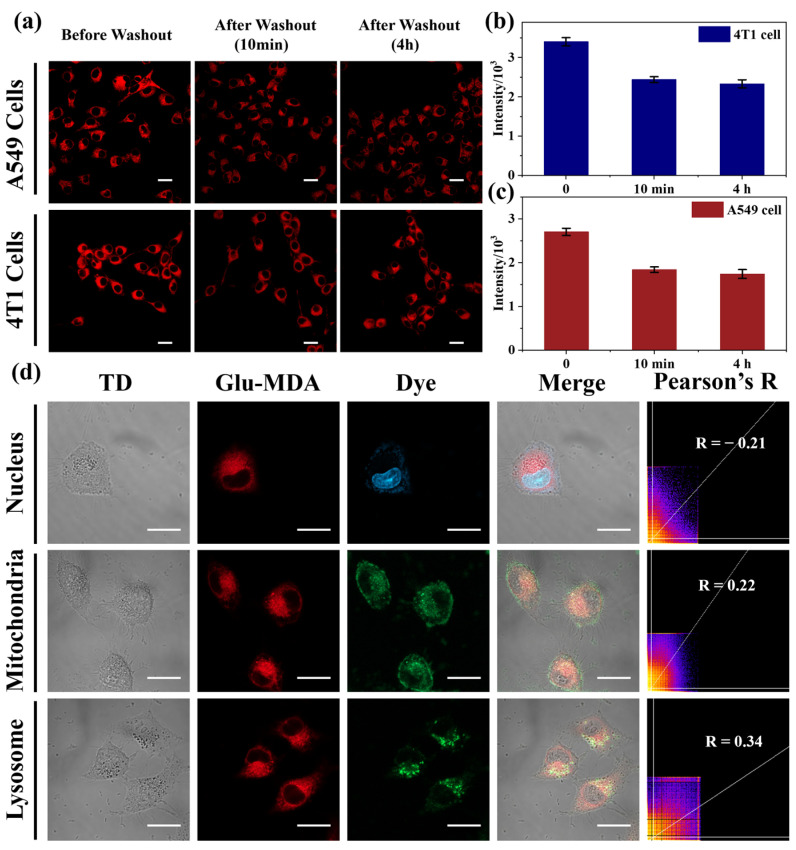
(**a**) Confocal images of A549 cells and 4T1 cells treated with 5 μM of Glu-MDA, before washing (left), after washing and incubated with medium for 10 min (center), after washing and incubated with medium for 4 h (right). λ_ex_ = 458 nm, red channel (490–600 nm). Scale bar = 20 μm. Fluorescence intensity of (**b**) A549 and (**c**) 4T1 cells before and after washing. Data are presented as mean ± standard deviation (SD) (n = 3). (**d**) Co-staining cell imaging of Glu-MDA (10 μM) with DAPI, Mito-Tracker Green and Lyso-Tracker Green in A549 cells. Scale bar = 20 μm.

The subsequent phase of the study investigated the intracellular retention of fluorescent reaction products generated by the probe Glu-MDA during the detection of GGT in cells. A549 cells and 4T1 cells were selected as the experimental model [43]. The cells were incubated with a 5 μM concentration of the Glu-MDA probe in cell culture medium for 10 min at 37 °C. Following this, fluorescence microscopy was employed to capture images of the cells before and after the washing step. As shown in Figure 4a–c, the fluorescence intensity of the A549 cells and 4T1 cells decreased slightly after the washing procedure. The decrease in fluorescence intensity after washing can be attributed to the exchange of some fluorophores, which are released during the enzymatic reaction, with the external environment. Furthermore, the re-incubation period in the probe-free medium was extended to 4 h to monitor alterations in intracellular fluorescence intensity. It was observed that the fluorescence intensity remained relatively stable compared to the level recorded immediately after the washing step (Figure 4a–c). These findings demonstrate that Glu-MDA effectively visualizes endogenous GGT activity in cells and exhibits good intracellular retention of its fluorescent reaction products.

### 2.13. Intracellular Product Distribution

In order to figure out the intracellular distribution of GGT-triggered probe Glu-MDA in tumor cells, a colocalization experiment of Glu-MDA-incubated A549 cells with 4′,6-diamidino-2-phenylindole (DAPI), the mitochondrial tracker (Mito-Tracker Green), and the lysosomal tracker (Lyso-Tracker Green) was then carried out [44]. It was observed that the DAPI channel exhibited a minimal overlap with the probe channel, and Mito-Tracker Green and Lyso-Tracker Green showed a partial overlap with the probe (Figure 4d). Furthermore, the colocalization coefficient (Pearson’s R) indicated that the fluorescence reaction products of the probe triggered by GGT were almost evenly distributed in various subcellular organelles within the cytoplasm rather than in the nucleus (Figure 4d). This finding indicates that upon Glu-MDA binding to the GGT binding pocket, the amide bond within Glu-MDA is cleaved by the active site of GGT. This process leads to the release of the fluorophore from the active pocket of GGT, followed by its diffusion into the cytoplasm.

### 2.14. Test Strip Experiment

**Figure 5:**
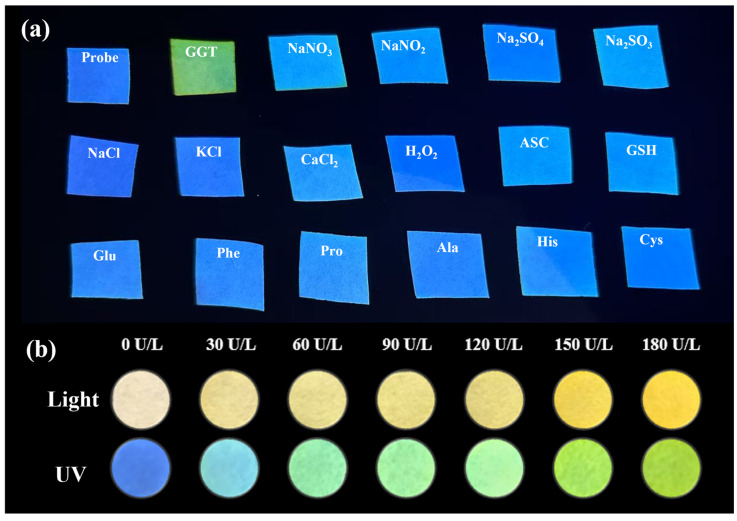
(**a**) The photograph of the Glu-MDA-coated filter papers with the different analytes under a 365 nm UV lamp. (**b**) Response of the Glu-MDA-coated filter papers to different concentrations of GGT (0–180 U/L) under a 365 nm UV lamp and under natural light.

In view of the favorable response exhibited by the probe Glu-MDA to GGT, a test strip for the pre-stained probe Glu-MDA was prepared by means of a dip-dyeing experiment. By applying diverse analytes to the test strip, the color changes in the test strip under both natural and ultraviolet light were observed. As shown in Figure 5a and Figure S34, the application of GGT to the filter paper demonstrated that it was the only analyte capable of inducing a significant color change. The color changed from white to yellow under natural light, and the blue fluorescence changed to green fluorescence under the irradiation of a 365 nm ultraviolet lamp (Figure 5a and Figure S34). The response of the test strip to different concentrations of GGT was further discussed. The test strip was immersed in a 1 mM Glu-MDA solution, and the dipped filter paper was then exposed to a GGT solution ranging from 0 to 180 U/L. Under both natural and 365 nm ultraviolet light, a distinct color change was observed on the test strip when the GGT activity exceeded 30 U/L, accompanied by a gradual increase in fluorescence intensity at higher GGT concentrations (Figure 5b and Figure S35). Furthermore, an evaluation was carried out to determine the fluorescence intensity of the pre-stained test strip after storage, as well as its ability to detect GGT. As shown in Figure S36a, the fluorescence intensity remained consistently stable for two days following the preparation of the test strip. Upon exposure to GGT, the test strip exhibited clear fluorescence color changes, and the fluorescence signal remained steady over time with negligible attenuation (Figure S36b). These findings conclusively demonstrate that the fluorescence of Glu-MDA pre-stained test paper exhibits good stability both prior to and following GGT detection. The test strip method has been shown to provide a portable and rapid means of detecting GGT using the probe Glu-MDA.

## 3. Materials and Methods

### 3.1. Materials and Instruments

Unless otherwise noted, all chemicals were obtained from commercial suppliers and used without further purification. ^1^H NMR and ^13^C NMR spectra were recorded on a Bruker AVANCE NEO 400 MHz or a Bruker AVANCE NEO 600 MHz NMR spectrometer. High-resolution mass spectra (HRMS) were obtained on an autoflex max MALDI TOF mass spectrometer (Bruker Daltonik GmbH, Bremen, Germany) or a HPLC-ESI-TOF/MS, Agilent G6224A (Agilent, Waldbronn, Germany) spectrometer. HPLC was performed on an Anters-1200 system with a C18, 5 μm HPLC Column (L × I.D., 15 cm × 4.6 mm). Condition: eluent, H_2_O/MeOH (*v*/*v*, 80/20); flow rate, 1 mL/min; temperature, 25 °C; injection volume, 10.0 μL. UV–visible absorption spectra were recorded on a UV-2600 spectrometer. Fluorescence spectra were carried out on an FLS1000 fluorescence spectrometer. Confocal fluorescence imaging was performed on Olympus FV1000 confocal microscopy or Zeiss Confocal LSM 980 Microscopy. The Accuri C6 plus flow cytometer was used to sort out specific cells. The detection limit and cytotoxicity were tested with a SpectraMax M2/M2e multifunctional microplate reader (Molecular Devices, San Jose, CA, USA).

### 3.2. Synthesis of Glu-MDA

Synthesis of compound 1. (R)-5-(tert-butoxy)-4-((tert-butoxycarbonyl) amino)-5-oxopenta-noic acid (900 mg, 3 mmol) was dissolved in 10 mL of redistilled DCM at 0 °C with thorough stirring to ensure complete dissolution. 1-(3-Dimethylaminopropyl)-3-ethylcarbodiimide hydrochloride (EDCI) (600 mg, 3 mmol) was added and activated for a period of 20 min, after which another 4-aminophenyl methanol (370 mg, 3 mmol) was dissolved in 10 mL of anhydrous DCM and dissolved by sonication. Subsequently, the substance was introduced to the reaction system at a slow rate and stirred at room temperature for an hour and a half. TLC monitored the reaction. The solvent was removed under reduced pressure, and the resulting solution was purified by column chromatography. Petroleum ether was then added, and the resulting precipitate was filtered and dried under vacuum to give compound 1 (yield: 91%). ^1^H NMR (400 MHz, CDCl_3_) δ 8.93 (s, 1H), 7.41 (d, J = 7.7 Hz, 2H), 7.16 (d, J = 7.8 Hz, 2H), 5.53 (d, J = 7.8 Hz, 1H), 4.51 (s, 2H), 4.08 (m, 1H), 2.33 (t, J = 6.4 Hz, 2H), 2.20–1.99 (m, 1H), 1.91–1.75 (m, 1H), 1.37 (s, 9H), 1.36 (s, 9H). ^13^C NMR (151 MHz, CDCl_3_) δ 186.39, 171.22, 170.77, 162.69, 156.67, 149.83, 145.58, 145.57, 138.86, 134.73, 134.25, 131.60, 130.68, 128.47, 128.37, 119.93, 114.38, 106.76, 101.09, 82.82, 80.56, 70.65, 53.19, 34.18, 30.76, 28.32, 27.98. MALDI-TOF/MS *m*/*z*: [M+Na] calculated for [C_21_H_32_N_2_NaO_6_]^+^ 431.2153, found 431.2162.

Synthesis of compound 2. A solution of compound 1 (1.8 g, 4.5 mmol) in anhydrous tetrahydrofuran (25 mL) was prepared, and PBr_3_ (600 μL, 6.5 mmol) was added dropwise at 0 °C with stirring and monitoring of the reaction at TLC. Upon completion of the reaction, the mixture was transferred to a 5% sodium bicarbonate solution (150 mL) and extracted with DCM (3 × 105 mL) three times. The organic layer was dried with sodium sulfate, the organic solvent was removed by evaporation, and purification was conducted via column chromatography. The resulting product was added to an adequate quantity of petroleum ether, which resulted in the precipitation of a solid. This solid was then filtered and dried, thereby yielding compound 2 (yield: 50%). ^1^H NMR (400 MHz, CDCl_3_) δ 9.10 (s, 1H), 7.62 (d, J = 8.0 Hz, 2H), 7.36 (d, J = 8.5 Hz, 2H), 5.38 (d, J = 7.8 Hz, 1H), 4.50 (s, 2H), 4.22 (t, J = 7.6 Hz, 1H), 2.44 (t, J = 6.5 Hz, 2H), 2.35–2.19 (m, 1H), 1.92–1.76 (m, 1H), 1.48 (s, 9H), 1.46 (s, 9H). MALDI-TOF/MS *m*/*z*: [M+Na] calculated for [C_21_H_31_BrN_2_NaO_5_]^+^ 493.1314, found 493.1291.

Synthesis of compound 3. 6-bromo-1H, 3H-benzo [de] isochromene-1,3-dione (1 g, 3.61 mmol) and prop-2-yn-1-amine (0.216 g, 4 mmol) were dissolved in a 100 mL three-necked flask, and 20 mL of ethanol was added and refluxed at 70 °C in an argon atmosphere. The reaction was detected by TLC. At the end of the reaction, the mixture was cooled to room temperature and then poured into water (50 mL) to obtain the precipitate. The precipitate was washed with water and dried to obtain product 3 (Yield: 82%). ^1^H NMR (400 MHz, DMSO-*d_6_*) δ 8.56 (d, J = 7.1 Hz, 1H), 8.51 (d, J = 8.1 Hz, 1H), 8.32 (d, J = 7.7 Hz, 1H), 8.19 (d, J = 7.7 Hz, 1H), 7.97 (t, J = 7.7 Hz, 1H), 4.77 (s, 2H), 3.19 (s, 1H).

Synthesis of MDA. Compound 3 (4.0 g, 12.73 mmol), N-hydroxysuccinimide (1.61 g, 14.01 mmol), and potassium carbonate (5.81 g, 42.02 mmol) were dissolved in DMSO (60 mL) and stirred at 80 °C for 3 h in an argon atmosphere. At the end of the reaction, it was cooled to room temperature, and diluted hydrochloric acid (1 M) was added to adjust the pH to 3. After precipitation, filtration, and washing, an appropriate amount of DCM was added to dissolve, and the product MDA was purified by column chromatography (yield: 79%). ^1^H NMR (400 MHz, DMSO-*d_6_*) δ 11.97 (s, 1H), 8.53 (d, J = 8.3 Hz, 1H), 8.47 (d, J = 7.2 Hz, 1H), 8.35 (d, J = 8.3 Hz, 1H), 7.75 (t, J = 7.8 Hz, 1H), 7.15 (d, J = 8.2 Hz, 1H), 4.74 (d, J = 2.2 Hz, 2H), 3.11 (t, J = 2.2 Hz, 1H). HPLC-ESI-TOF/MS *m*/*z*: [M-H] calculated for [C_15_H_8_NO_3_]^−^ 250.2335, found 250.0513.

Synthesis of Boc-Glu-MDA. MDA (110 mg, 0.44 mmol), compound 2 (245 mg, 0.52 mmol), and anhydrous K_2_CO_3_ (200 mg) were added to 5 mL of anhydrous DMF. The mixture was then stirred at room temperature for 12 h. Following this, the mixture was extracted with ethyl acetate, dried with anhydrous sodium sulphate, and the volatile solvent was evaporated. The final purification stage was conducted by column chromatography, resulting in the formation of the final yellow product, Boc-Glu-MDA (yield: 61%). ^1^H NMR (400 MHz, DMSO-*d*_6_) δ 10.01 (s, 1H), 8.48 (t, J = 8.3 Hz, 2H), 8.42 (d, J = 8.2 Hz, 1H), 7.77 (t, J = 7.8 Hz, 1H), 7.66 (d, J = 8.0 Hz, 2H), 7.52 (d, J = 8.0 Hz, 2H), 7.39 (d, J = 8.4 Hz, 1H), 7.17 (d, J = 7.6 Hz, 1H), 5.37 (s, 2H), 4.75 (s, 2H), 3.96–3.71 (m, 1H), 3.14 (s, 1H), 2.42 (t, J = 6.7 Hz, 2H), 2.01 (m, 1H), 1.81 (m, 1H), 1.40 (s, 9H), 1.38 (s, 9H). ^13^C NMR (151 MHz, DMSO-*d*_6_) δ 172.09, 170.91, 163.27, 162.54, 160.10, 156.01, 139.82, 133.97, 131.81, 130.67, 129.30, 129.15, 129.00, 126.95, 123.40, 121.91, 119.52, 114.27, 107.94, 80.80, 79.98, 78.54, 73.33, 70.86, 54.34, 33.15, 29.38, 28.65, 28.12, 26.64. HPLC-ESI-TOF/MS *m*/*z*: [M+Na] calculated for [C_36_H_39_N_3_NaO_8_]^+^ 664.2635, found 664.2636.

Synthesis of Glu-MDA. Boc-Glu-MDA (100 mg, 0.33 mmol) was added to 2 mL of anhydrous DCM at 0 °C. Subsequently, 2 mL of TFA was added dropwise, and the solution was stirred for 2 h. Then, ether was added until no further solid precipitate was observed. The solution was filtered, washed, and dried to obtain the final product, Glu-MDA (yield: 54%). ^1^H NMR (400 MHz, DMSO-*d*_6_/CF_3_COOD (30:1 *v*/*v*)) δ 10.16 (s, 1H), 8.56–8.48 (m, 2H), 8.46 (d, J = 7.3 Hz, 1H), 8.32 (m, 3H), 7.80 (m, 1H), 7.66 (m, 2H), 7.53 (d, J = 5.7 Hz, 2H), 7.42 (d, J = 7.1 Hz, 1H), 5.40 (s, 2H), 4.76 (s, 2H), 3.99 (m, 1H), 3.14 (s, 1H), 2.58 (m, 2H), 2.10 (m, 2H). ^13^C NMR (151 MHz, DMSO-*d*_6_/CF_3_COOD (30:1 *v*/*v*)) δ 171.34, 170.35, 163.31, 162.58, 160.12, 139.64, 134.01, 131.87, 130.91, 129.31, 129.14, 129.07, 127.01, 123.46, 121.98, 119.69, 114.35, 108.02, 79.98, 73.33, 70.83, 52.02, 31.96, 29.39, 26.06. HPLC-ESI-TOF/MS *m*/*z*: [M-H] calculated for [C_27_H_22_N_3_O_6_]^−^ 484.1514, found 484.1512.

### 3.3. Molecular Docking Experiment

The molecular docking simulation of the fluorescent probe Glu-MDA and GGT was performed using AutoDock 4.2 software. The crystal structure of GGT (PDB: 4GDX) was obtained from the Protein Data Bank. During the simulation process, GGT was designed as a rigid receptor structure, and Glu-MDA was designed as a flexible ligand structure, with the two structures being semi-flexibly docked. The parameters of the GGT crystal were adjusted by removing water molecules, adding hydrogen atoms, and assigning Gasteiger charges to the ligand and fragment. It is important to note that the probe Glu-MDA can rotate during the docking process. The docking box was constructed to cover the potential binding region, and the optimal binding site, orientation, and conformation of the ligand within this box were determined using the Lamarck GA (4.2) method. Glu-MDA was subjected to 200 independent docking experiments.

### 3.4. General Procedure for GGT Detection

The fluorophore MDA stock solution (3 mM) and the probe Glu-MDA stock solution (3 mM) were prepared in DMSO, and the GGT stock solution (2 U/L), DON stock solution (10 mM), and GGsTop stock solution (10 mM) were prepared in ultrapure water. The stock solutions of NaNO_3_, NaNO_2_, Na_2_SO_4_, Na_2_SO_3_, NaCl, KCl, CaCl_2_, H_2_O_2_, L-ascorbic acid, Phe, Pro, Ala, His, Cys, Hcy, GSH, GR, GSH-Px, GST, and GCL were prepared in ultrapure water. Selectivity and anti-interference tests were then carried out. The concentration of the enzyme stock solution was 1 U/mL, and the concentration of the other stock solutions was 10 mM, unless otherwise indicated. Unless indicated otherwise and without further elaboration, all UV–vis and fluorescence spectra were conducted in a 1:4 (*v*/*v*) mixture of DMSO and PBS (10 μM, pH 7.4), with all solutions being equilibrated for a duration of 20 min prior to analysis.

### 3.5. Determination of the Detection Limit

The probe Glu-MDA (10 μM) was incubated with varying concentrations of GGT samples for 5 min under controlled conditions. Three groups of parallel samples and ten blank controls were set up in the experiment, and then the fluorescence response was quantitatively analyzed with a SpectraMax M2/M2e multifunctional microplate reader (λ_ex_ = 450 nm and λ_em_ = 550 nm). The detection limit was subsequently calculated according to the following equation:

LOD = 3*σ*/*k*, (1)

where *σ* is the standard deviation of blank measurements, *k* is the slope of the linear regression equation, and LOD is the detection limit.

### 3.6. Cell Culture

The cultivation of 3T3 cells, LX-2 cells, A549 cells, 4T1 cells, and HepG2 cells was performed in Dulbecco’s Modified Eagle Medium (DMEM). This medium contained 1% penicillin–streptomycin solution and 10% fetal bovine serum. The culture bottles were incubated at 37 °C with 5% CO_2_.

### 3.7. Cytotoxicity Assay

3T3 cells, A549 cells, and 4T1 cells were seeded in 96-well plates at a density of 5 × 10^3^ cells/well and incubated for 24 h. Thereafter, the cells were treated with Glu-MDA at concentrations of 0, 2.5, 5, 10, 20, and 40 μM, respectively. Following a further 24 h of incubation, the cells were treated with 0.5 mg/mL of MTT and incubated for a further 4 h. Following the removal of the medium, DMSO was added, and then the absorbance at 570 nm was measured on a SpectraMax M2/M2e multifunctional microplate reader.

### 3.8. Cell Imaging

In order to explore the concentration-dependent effects of the probe Glu-MDA and perform cell imaging experiments, LX-2 cells, 3T3 cells, HepG2 cells, A549 cells, and 4T1 cells were incubated with cell culture medium containing different concentrations of Glu-MDA (0, 0.5, 1, 2.5, and 5 μM) in laser confocal dishes for 5 min (37 °C, 5% CO_2_). Following the completion of the incubation period, the samples were subjected to two washes with PBS. Thereafter, 1 mL of PBS was added to the samples. Fluorescence cell imaging was performed on an Olympus FV1000 confocal microscope using a laser source at a wavelength of 458 nm. The fluorescence signal of the cells was collected under a 60× oil immersion lens and within the range of 490–600 nm.

In the experiment to explore the optimal incubation time, the A549 cells and 4T1 cells were incubated with cell culture medium containing 2.5 μM of Glu-MDA in a laser confocal dish for 0, 1, 3, 5, and 10 min (37 °C, 5% CO_2_), respectively. After the incubation period was completed, the samples were washed twice with PBS. Thereafter, 1 mL of PBS was added to the samples. Fluorescence cell imaging was performed on an Olympus FV1000 confocal microscope with a laser source at a wavelength of 458 nm. The fluorescence signal of the cells was collected under a 60× oil immersion lens and within the range of 490–600 nm.

The HepG2 cells were subjected to an incubation process, after which they were seeded in a laser confocal dish and divided into four distinct groups. In the first group, cells were exposed to PBS buffer for 5 min. In the second group, cells were incubated with Glu-MDA (2.5 μM) for 5 min. In the third group, cells were pretreated with DON (1 mM) for 30 min, followed by incubation with Glu-MDA (2.5 μM) for 5 min. In the fourth group, cells were pretreated with GGsTop (1 mM) for 30 min and subsequently incubated with Glu-MDA (2.5 μM) for 5 min. Fluorescence cell imaging was performed on an Olympus FV1000 confocal microscope with a laser source at a wavelength of 458 nm. The fluorescence signal of the cells was collected under a 60× oil immersion lens and within the range of 490–600 nm.

### 3.9. Flow Cytometry Experiment

A549 cells were dispersed in culture flasks and cultured at 37 °C in an atmosphere of 5% CO_2_ and 95% air for 24 h. Subsequently, the cells were washed with PBS, and the cell culture medium containing a 5 μM Glu-MDA was added to each culture flask, followed by incubation for 2, 5, 10, and 20 min, respectively. Concurrently, the control blank group was not subjected to any treatment. The cells were washed with PBS, detached from the culture flasks by a Trypsin-EDTA solution, centrifuged at 1500 rpm for 5 min, resuspended in PBS, and filtered through a 40 μm filter screen for flow cytometric analysis using an Accuri C6 plus flow cytometer (λ_ex_ = 488 nm and FITC 533/30 nm).

### 3.10. Detection of GGT Under External Stimulation

The probe Glu-MDA was utilized to detect the endogenous GGT activity under external stimulation. In order to produce different levels of GGT, the cells were pretreated with cell culture medium containing 2.5 mM NaBu for different periods of time (0, 4, 8, and 12 h). Subsequent to pretreatment, 5 μM of the probe Glu-MDA was added to the medium and incubated at 37 °C, 5% CO_2_ for 10 min. After two washes with PBS, 1 mL of PBS was added. Fluorescence cell imaging was performed on an Olympus FV1000 confocal microscope with a laser source at a wavelength of 458 nm. The fluorescence signal of the cells was collected under a 60× oil immersion lens and within the range of 490–600 nm.

### 3.11. Cell Retention of Fluorescent Reaction Products

A549 cells and 4T1 cells were divided into three groups and incubated with cell culture medium containing 5 μM of Glu-MDA in a laser confocal dish for 10 min at 37 °C with 5% CO_2_. After the first incubation, cells were washed twice with PBS, and 1 mL of PBS was added. For the second incubation, cells were washed twice with PBS, medium was added, and the samples were incubated for 10 min. After this incubation period, the medium was removed, and 1 mL of PBS was added. For the third incubation, cells were washed twice with PBS, medium was added, and the samples were incubated for 4 h. After this incubation period, the medium was removed, and 1 mL of PBS was added. The three groups of cells were imaged on an Olympus FV1000 confocal microscope with a laser source at a wavelength of 458 nm, and the fluorescence signals of the cells were collected under a 60× oil lens within the range of 490–600 nm.

### 3.12. Fluorescence Reaction Product Distribution Experiment

A549 cells were cultured at 37 °C for 24 h under normoxic conditions. A549 cells were treated with 5 μM of the probe Glu-MDA for 5 min and then co-incubated with 2 μg/mL of DAPI, 0.05 μM of Mito-Tracker Green, and 0.05 μM of Lyso-Tracker Green at 37 °C for 10 min. Under 405 nm excitation, DAPI stains emit blue fluorescence in the range of 410–460 nm. Under 488 nm excitation, Mito-Tracker and Lyso-Tracker dyes emit green fluorescence in the range of 500–530 nm. Under 458 nm excitation, the Glu-MDA probe emits fluorescence in the range of 560–600 nm. Fluorescence cell imaging was performed on a Zeiss Confocal LSM 980 microscope. The fluorescence signal of the cells was collected under a 100× oil immersion lens.

### 3.13. Test Strips Experiment

The Glu-MDA solution (1 mM) was prepared with DMSO, and the Glu-MDA solution was evenly dropped onto the test paper and dried in an oven at 60 °C for 30 min to prepare the probe strip.

## 4. Conclusions

In summary, we designed and synthesized a fluorescent probe Glu-MDA for detecting and imaging overexpressed GGT in tumor cells. The spatial steric hindrance was reduced by connecting the self-immolative short linker with the recognition group. GGT was utilized to excise the active site of the recognition group, thereby releasing the fluorophore MDA through a spontaneous dissociation process. This resulted in a rapid restoration of fluorescence at 550 nm. The probe was demonstrated to facilitate the rapid and efficient detection of GGT in cancer cells, which can be attributed to to its good selectivity, adequate photostability, and low detection limit (0.044 U/L). Eventually, the portable and rapid detection of GGT-containing solution samples was achieved from a macroscopic perspective using Glu-MDA pre-coloring test strips.

## Data Availability

Data are contained within the article and Supplementary Materials.

## Supplementary Materials

The following supporting information can be downloaded at: https://www.mdpi.com/article/10.3390/molecules30153174/s1, Figure S1: ^1^H NMR spectrum of 1; Figure S2: ^13^C NMR spectrum of 1; Figure S3: MALDI -TOF/MS of 1; Figure S4: ^1^H NMR spectrum of 2; Figure S5: MALDI -TOF/MS of 2; Figure S6: ^1^H NMR spectrum of 3; Figure S7: ^1^H NMR spectrum of MDA; Figure S8: HPLC-ESI-TOF/MS of MDA; Figure S9: ^1^H NMR spectrum of Boc-Glu-MDA; Figure S10: ^13^C NMR spectrum of Boc-Glu-MDA; Figure S11: HPLC-ESI-TOF/MS of Boc-Glu-MDA; Figure S12: ^1^H NMR spectrum of Glu-MDA; Figure S13: ^13^C NMR spectrum of Glu-MDA; Figure S14: HPLC-ESI-TOF/MS of Glu-MDA; Figure S15: The absorption curves of fluorophore MDA and probe Glu-MDA in different solvents; Figure S16: The normalized excitation and emission spectra of the fluorophore MDA and the probe Glu-MDA; Figure S17: The fluorescence spectra of probe Glu-MDA and fluorophore MDA; Figure S18: The absorption stability of fluorophore MDA in DMSO/PBS (1:4 *v*/*v*) mixed solution; Figure S19: Fluorescence stability of fluorophore MDA; Figure S20: The stability of fluorophore MDA under different pH; Figure S21: The linear change in absorbance of the probe Glu-MDA with GGT over time at 376 nm and 452 nm; Figure S22: Fluorescence kinetics of Glu-MDA on GGT; Figure S23: Selectivity and anti-interference of probe Glu-MDA; Figure S24: Cytotoxicity of probe Glu-MDA in different cells; Figure S25: Cell imaging of 3T3 cells treated with different concentrations of Glu-MDA; Figure S26: Cell imaging of LX-2 cells treated with different concentrations of Glu-MDA; Figure S27: Cell imaging of 4T1 cells treated with different concentrations of Glu-MDA; Figure S28: Cell imaging of A549 cells treated with different concentrations of Glu-MDA; Figure S29: Cell imaging of HepG2 cells treated with different concentrations of Glu-MDA; Figure S30: Cell imaging after incubation of 4T1 cells with probe Glu-MDA for different times; Figure S31: Cell imaging after incubation of A549 cells with probe Glu-MDA for different times; Figure S32: Detection of GGT in cells treated with NaBu for different times; Figure S33: The fluorescence intensity of Glu-MDA in HepG2 cells treated with NaBu for different times; Figure S34: The photograph of the probe Glu-MDA-coated filter strips with the different analytes under natural light; Figure S35: The response of Glu-MDA coated filter paper strips to different concentrations of GGT; Figure S36: The fluorescence stability of Glu-MDA pre-stained test strips at different concentrations of GGT.
