# Supplementary material for: Naphthalimide-Based Fluorescent Probe for Portable and Rapid Response to γ-Glutamyl Transpeptidase
Source: Molecules. 2025 Jul 29;30(15):3174. doi: 10.3390/molecules30153174 (PMC12348333; doi:10.3390/molecules30153174)
Supplement: Supplementary file 1 [file molecules-30-03174-s001.zip › molecules-3621283-supplementary.pdf]

Molecules

Supporting Information

Naphthalimide-Based Fluorescent Probe for Portable and Rapid Response to γ-Glutamyl Transpeptidase

Jinhu Wang [1], Xianchao Jia [1], Yihao Zhang [1], Ye Gao [1], Lei Zhang [1], Changgong Meng [1], Zhaohui Wang [2,] *and Yang Jiao [1,] *

[1] State Key Laboratory of Fine Chemical, School of Chemical Engineering, Dalian University of Technology, Dalian 116024, China; wjh937@mail.dlut.edu.cn (J.W.); xcjia@mail.dlut.edu.cn (X.J.); 1329731948@mail.dlut.edu.cn (Y.Z.); gaoye@mail.dlut.edu.cn (Y.G.); aizome@mail.dlut.edu.cn (L.Z.); cgmeng@dlut.edu.cn (C.M.); jiaoyang@dlut.edu.cn (Y.J.)

[2] Department of Gastroenterology, Central Hospital of Dalian University of Technology (Dalian Municipal Central Hospital), Dalian 116024, China; wdl2000411@aliyun.com (Z.W.)

* Correspondence: jiaoyang@dlut.edu.cn (Y.J.); wdl2000411@aliyun.com (Z.W.)

Contents

Figure S1. ^{1}H NMR spectrum of **1**
Figure S2. ^{13}C NMR spectrum of **1**
Figure S3. MALDI -TOF/MS of **1**
Figure S4. ^{1}H NMR spectrum of **2**
Figure S5. MALDI -TOF/MS of **2**
Figure S6. ^{1}H NMR spectrum of **3**
Figure S7. ^{1}H NMR spectrum of **MDA**
Figure S8. HPLC-ESI-TOF/MS of **MDA**
Figure S9. ^{1}H NMR spectrum of **Boc-Glu-MDA**
Figure S10. ^{13}C NMR spectrum of **Boc-Glu-MDA**
Figure S11. HPLC-ESI-TOF/MS of **Boc-Glu-MDA**
Figure S12. ^{1}H NMR spectrum of **Glu-MDA**
Figure S13. ^{13}C NMR spectrum of **Glu-MDA**
Figure S14. HPLC-ESI-TOF/MS of **Glu-MDA**
Figure S15. The absorption curves of fluorophore **MDA** and probe **Glu-MDA** in different solvents
Figure S16. The normalized excitation and emission spectra of the fluorophore **MDA** and the probe **Glu-MDA**
Figure S17. The fluorescence spectra of probe **Glu-MDA** and fluorophore **MDA**
Figure S18. The absorption stability of fluorophore **MDA** in DMSO/PBS (1: 4 *v/v*) mixed solution
Figure S19. Fluorescence stability of fluorophore **MDA**
Figure S20. The stability of fluorophore **MDA** under different pH
Figure S21. The linear change in absorbance of the probe **Glu-MDA** with **GGT** over time at 376 nm and 452 nm
Figure S22. Fluorescence kinetics of **Glu-MDA** on **GGT**
Figure S23. Selectivity and anti-interference of probe **Glu-MDA**
Figure S24. Cytotoxicity of probe **Glu-MDA** in different cells
Figure S25. Cell imaging of 3T3 cells treated with different concentrations of **Glu-MDA**
Figure S26. Cell imaging of LX-2 cells treated with different concentrations of **Glu-MDA**
Figure S27. Cell imaging of 4T1 cells treated with different concentrations of **Glu-MDA**
Figure S28. Cell imaging of A549 cells treated with different concentrations of **Glu-MDA**
Figure S29. Cell imaging of HepG2 cells treated with different concentrations of **Glu-MDA**
Figure S30. Cell imaging after incubation of 4T1 cells with **Glu-MDA** for different times
Figure S31. Cell imaging after incubation of A549 cells with **Glu-MDA** for different times
Figure S32. Detection of **GGT** in cells treated with NaBu for different times
Figure S33. The fluorescence intensity of **Glu-MDA** in HepG2 cells treated with NaBu for different times
Figure S34. The photograph of the probe **Glu-MDA**-coated filter strips with the different analytes under natural light
Figure S35. The response of **Glu-MDA** coated filter paper strips to different concentrations of **GGT**
Figure S36. The fluorescence stability of **Glu-MDA** pre-stained test strips at different concentrations of **GGT**

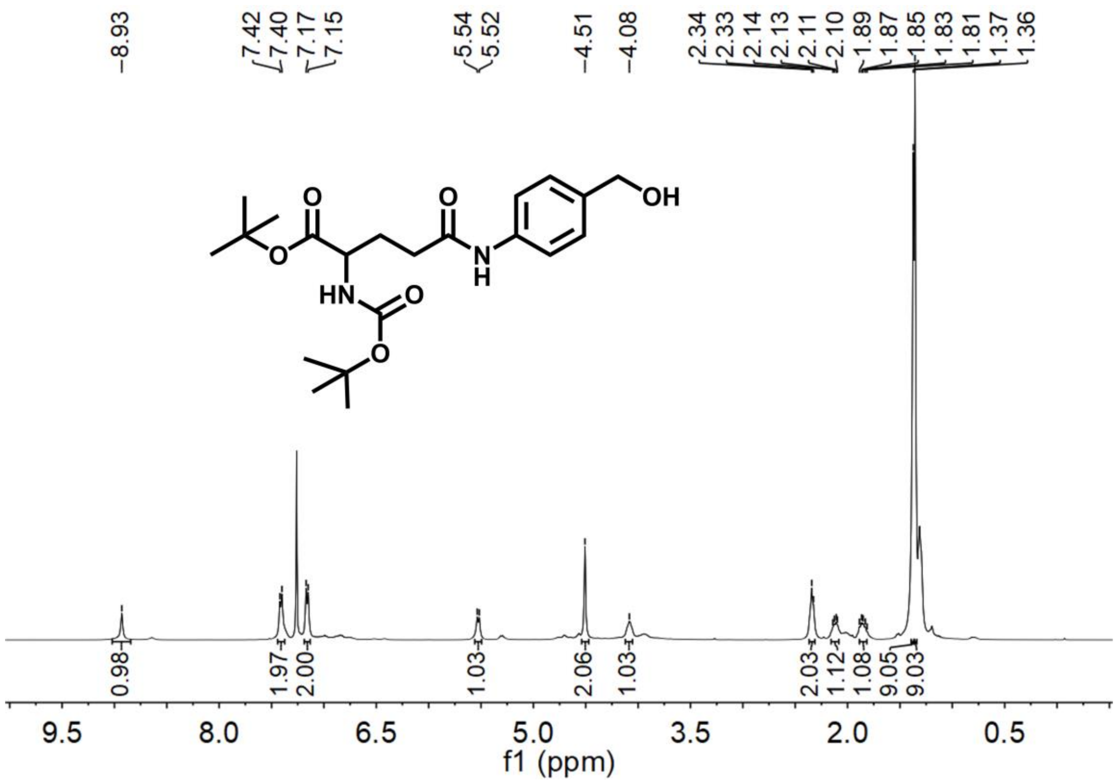

Figure S1. ^{1}H NMR spectrum of **1** in $CDCl_3$

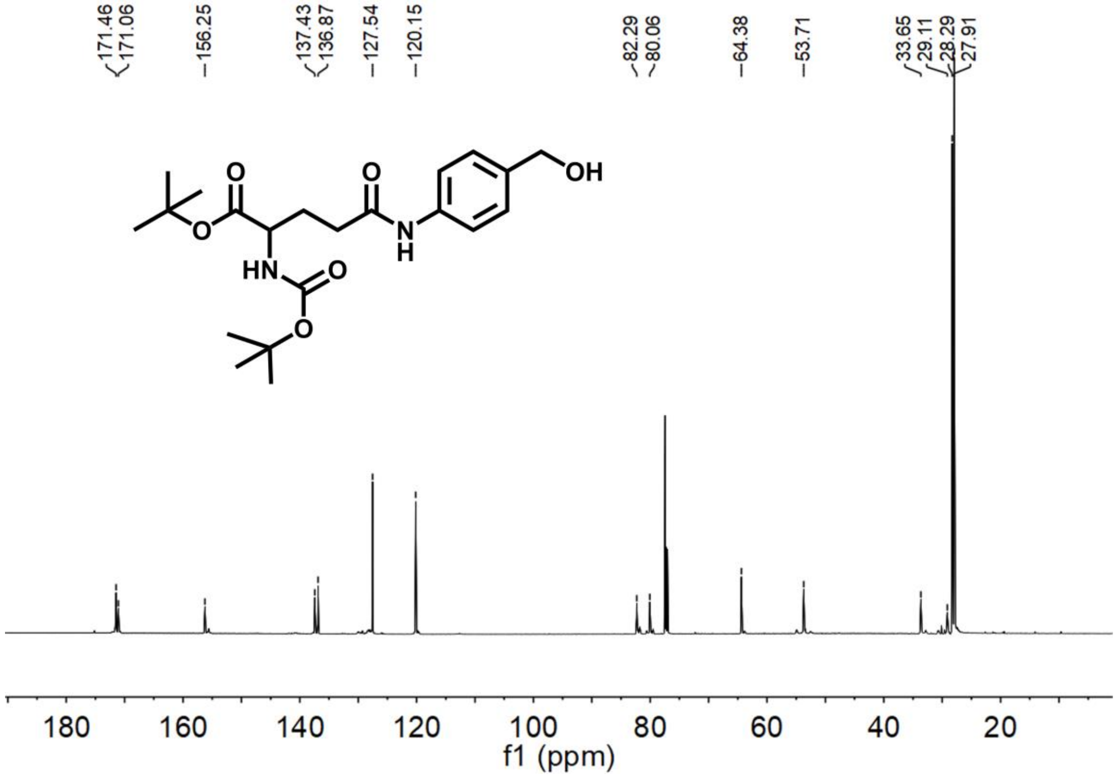

Figure S2. ^{13}C NMR spectrum of **1** in $CDCl_3$

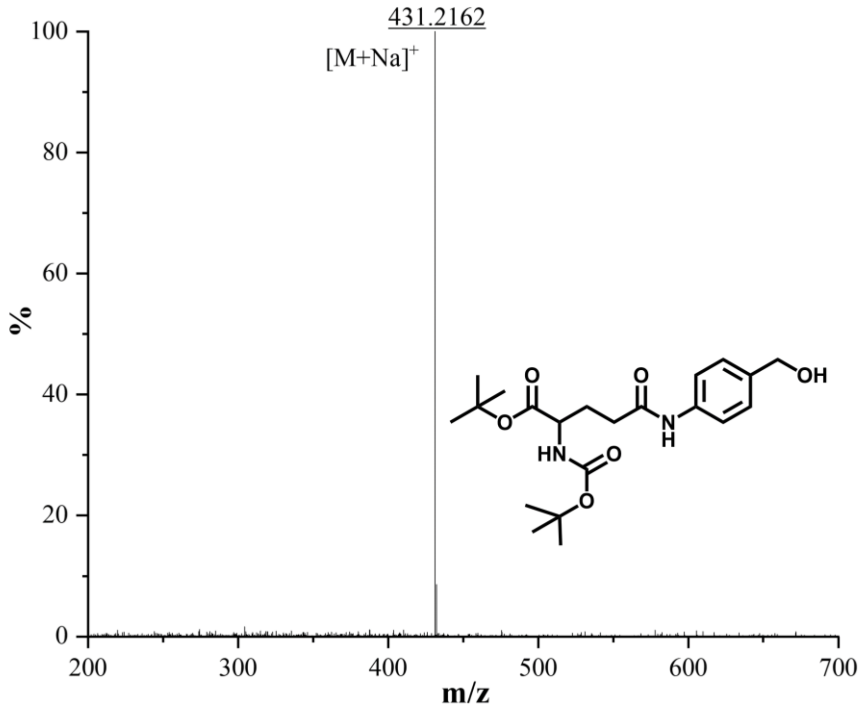

Figure S3. MALDI -TOF/MS of **1**

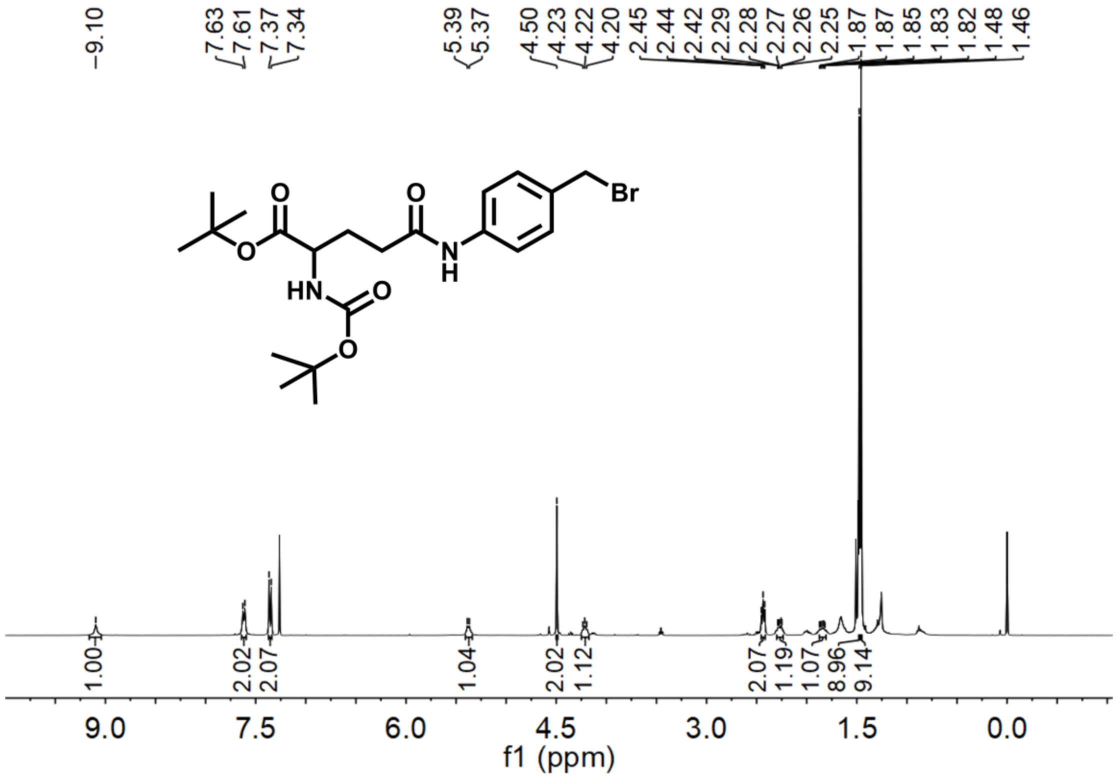

Figure S4. ^{1}H NMR spectrum of **2** in $CDCl_3$

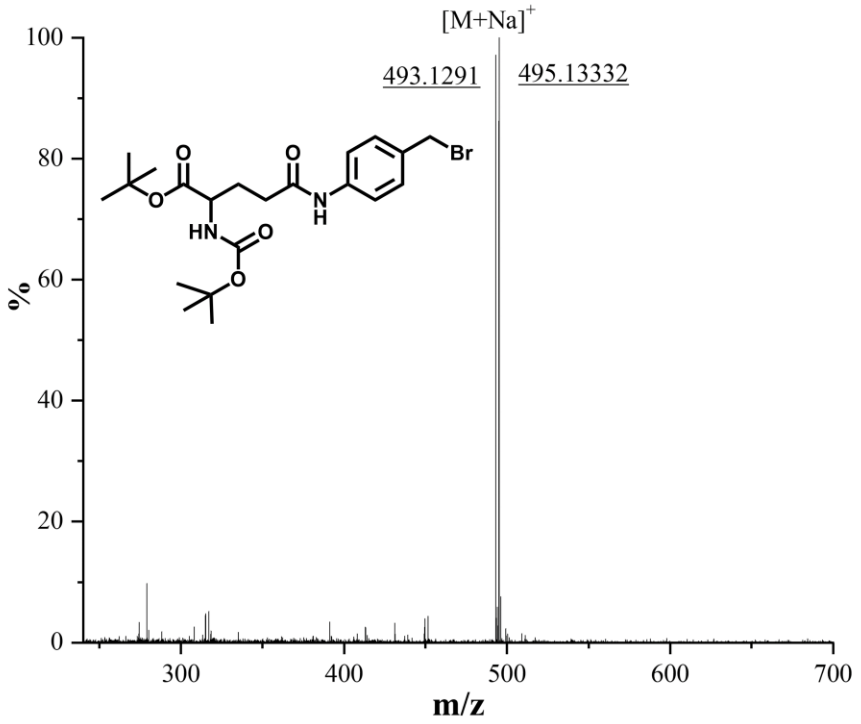

Figure S5. MALDI -TOF/MS of **2**

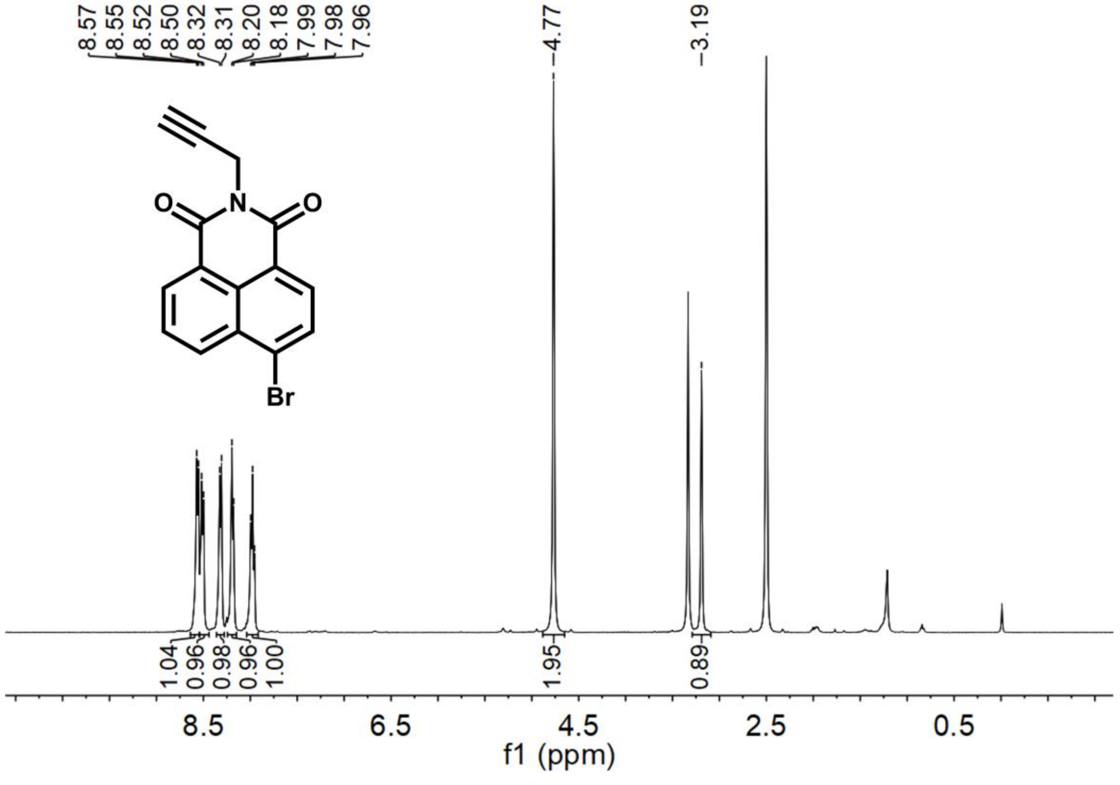

Figure S6. ^{1}H NMR spectrum of **3** in DMSO-d_6

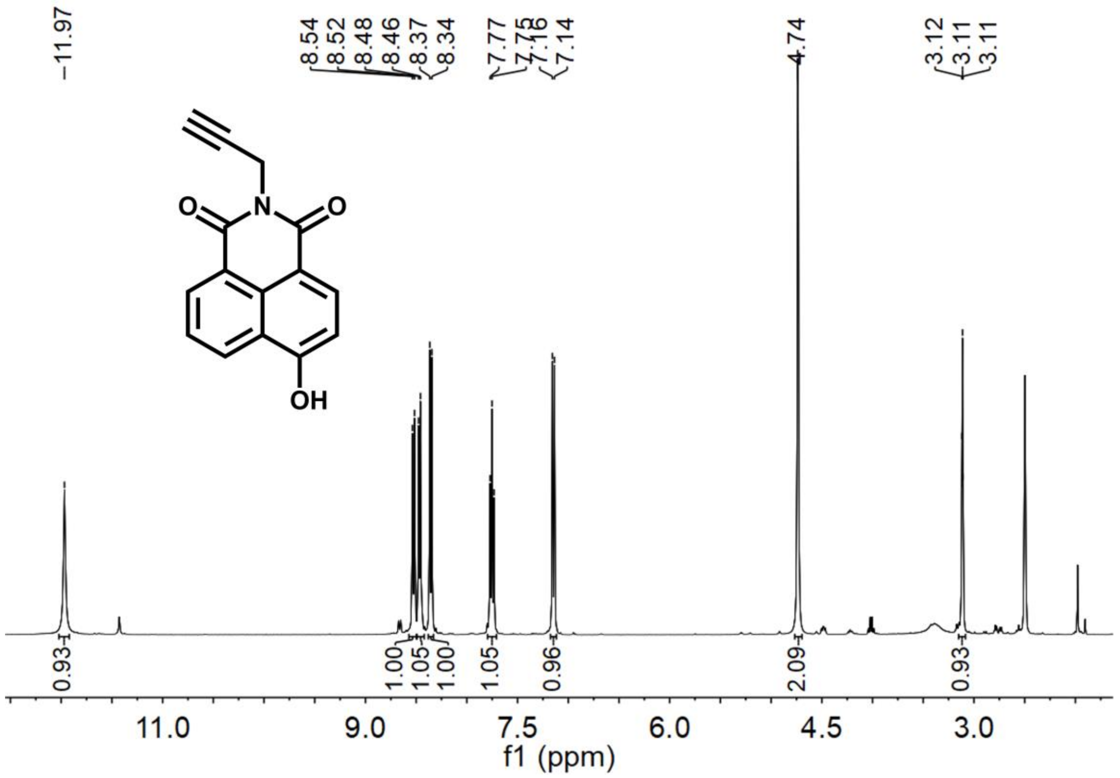

Figure S7. ^{1}H NMR spectrum of **MDA** in DMSO-d_6

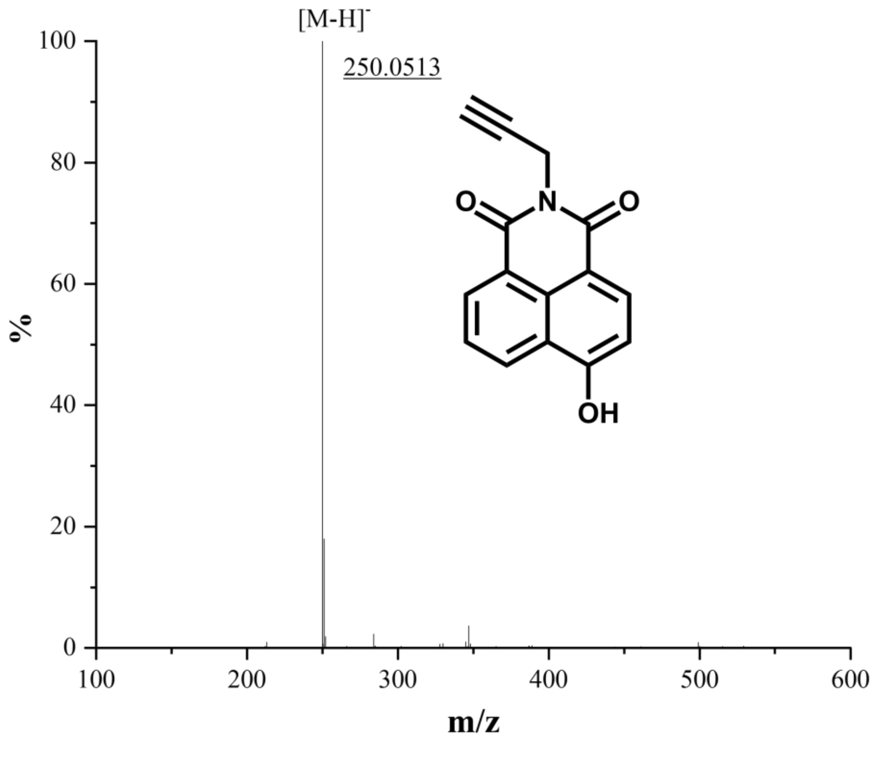

Figure S8. HPLC-ESI-TOF/MS of **MDA**

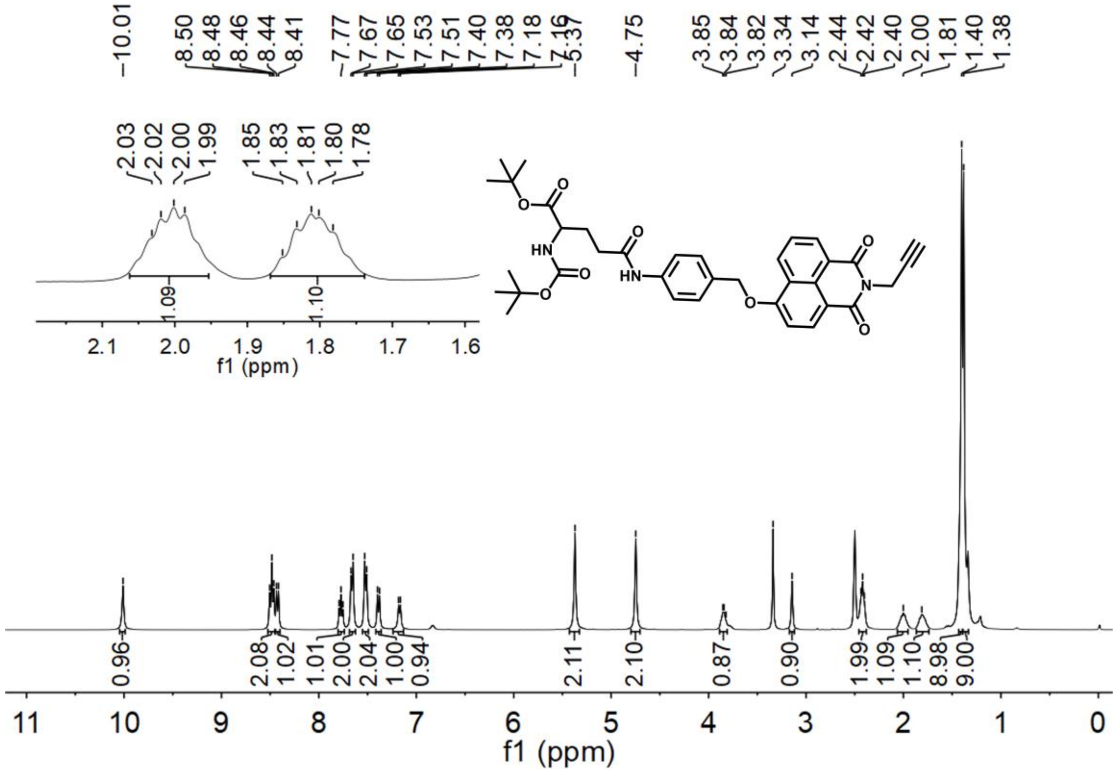

Figure S9. ^{1}H NMR spectrum of **Boc-Glu-MDA** in DMSO-d_6

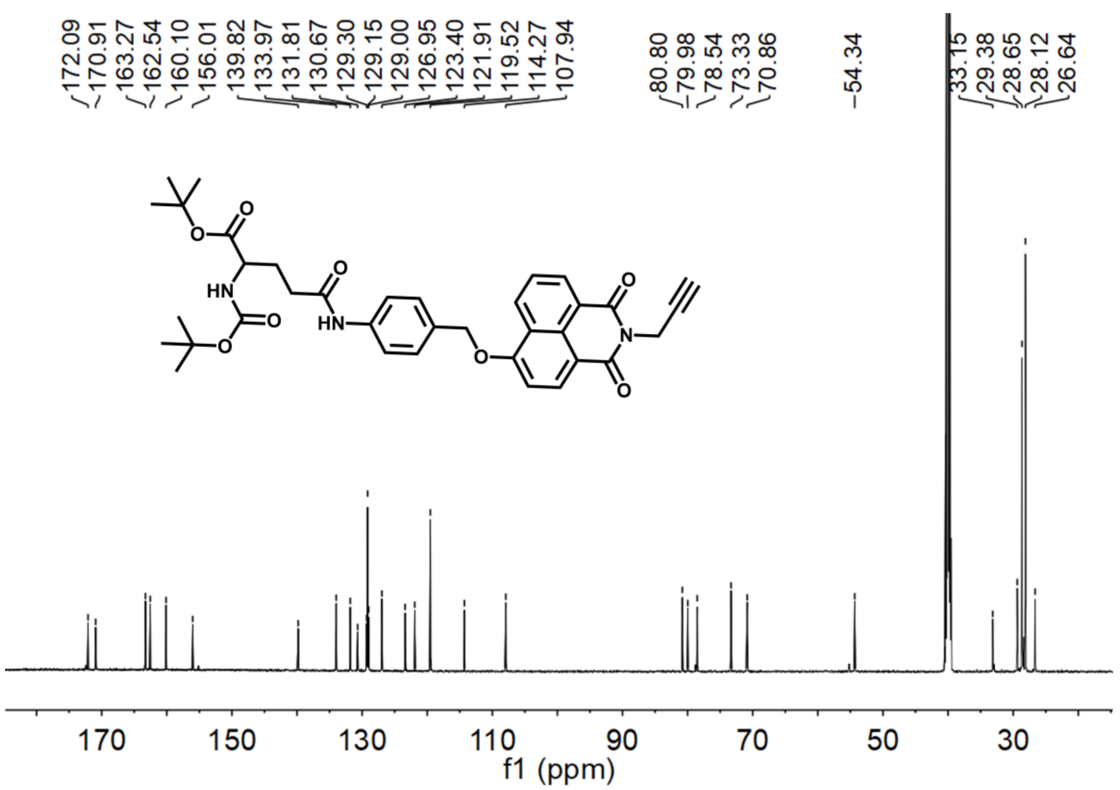

Figure S10. ^{13}C NMR spectrum of **Boc-Glu-MDA** in DMSO-d_6

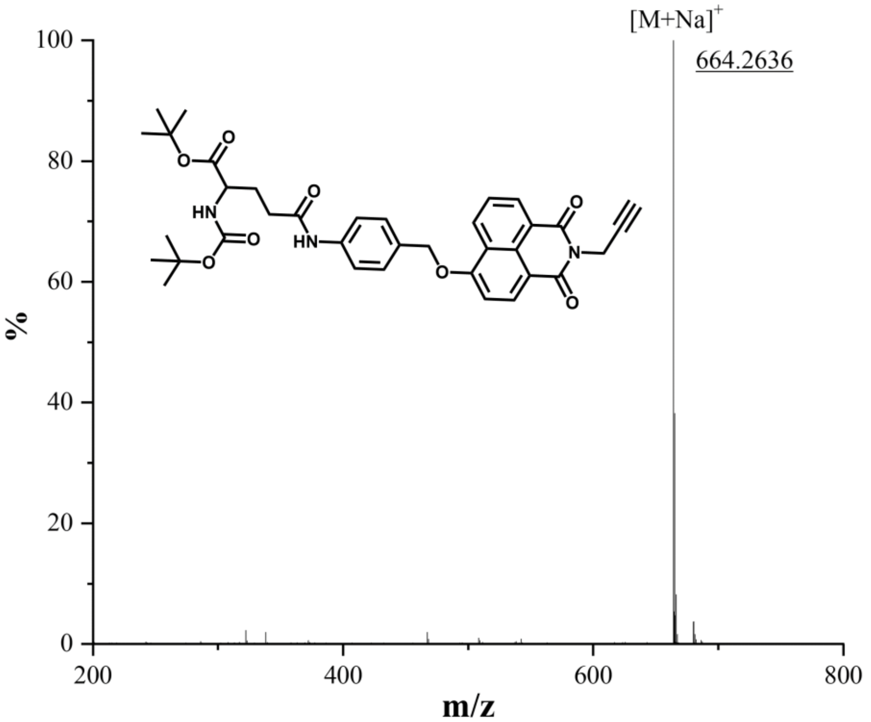

Figure S11. HPLC-ESI-TOF/MS of **Boc-Glu-MDA**

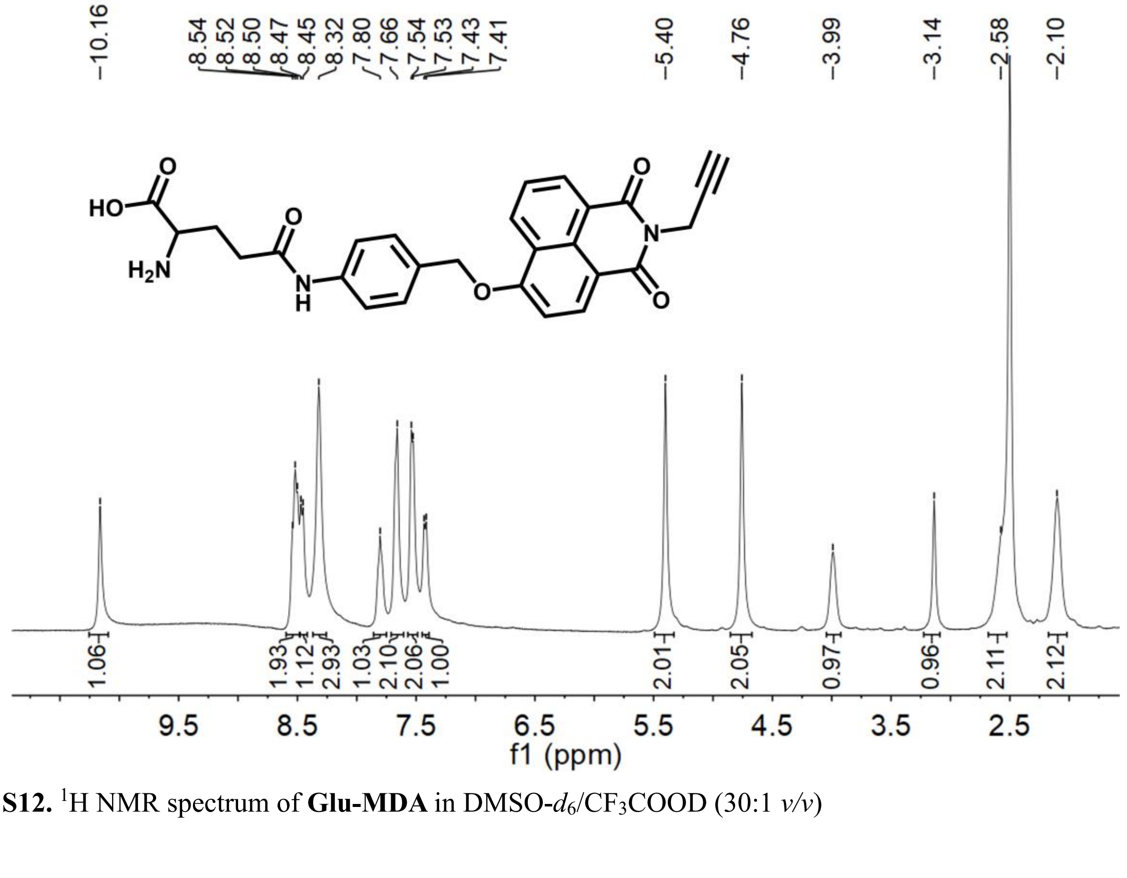

Figure S12. ^{1}H NMR spectrum of **Glu-MDA** in DMSO-d_6/CF_3COOD (30:1 *v/v*)

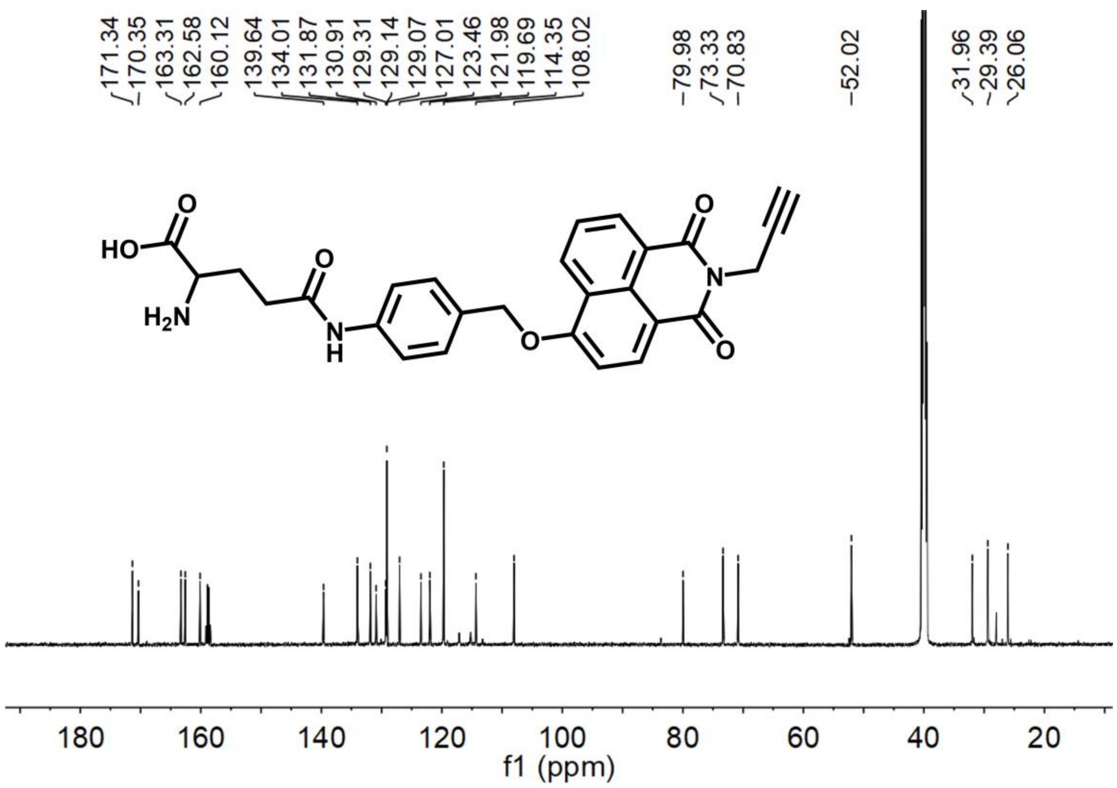

Figure S13. ^{13}C NMR spectrum of **Glu-MDA** in DMSO-d_6/CF_3COOD (30:1 *v/v*)

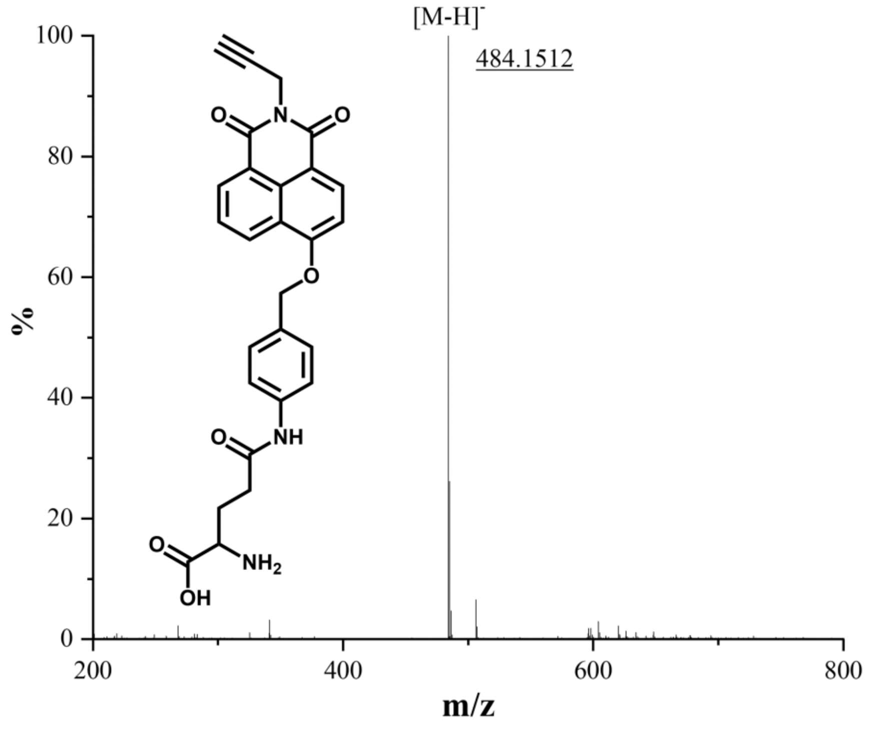

Figure S14. HPLC-ESI-TOF/MS of **Glu-MDA**

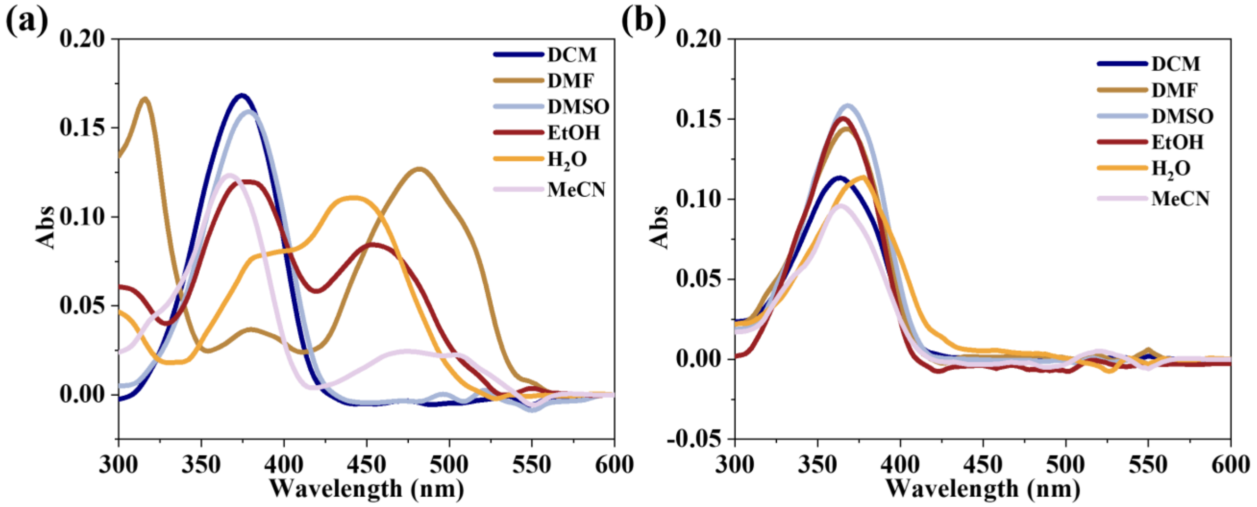

Figure S15. The absorption curves of (a) fluorophore **MDA** (10 μM) and (b) probe **Glu-MDA** (10 μM) in DCM, DMF, DMSO, EtOH, H_2O and MeCN

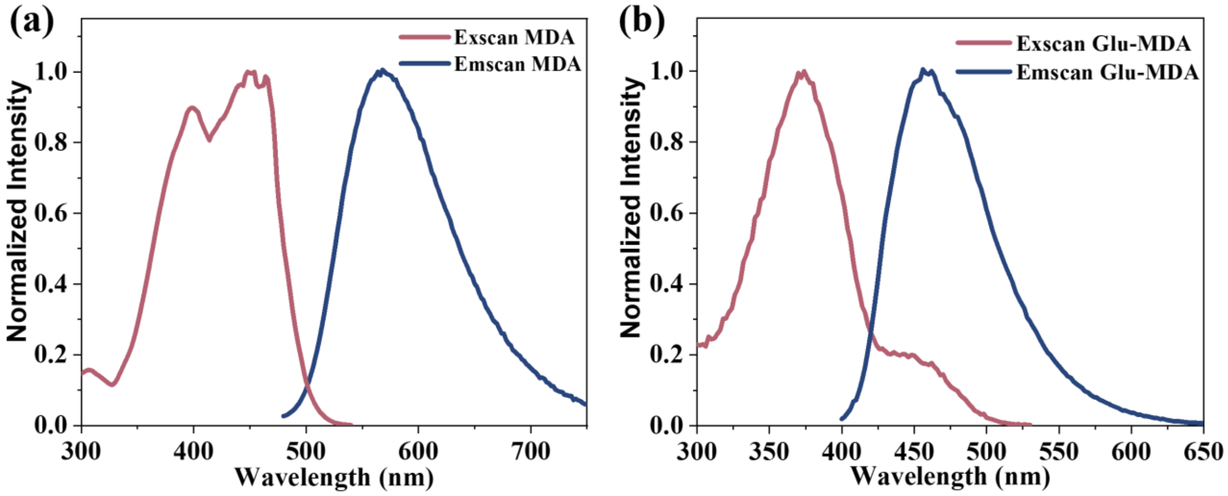

Figure S16. In DMSO/PBS (1: 4 *v/v*) mixed solution, (a) the normalized excitation and emission spectra of the fluorophore **MDA** (10 μM) and (b) the normalized excitation and emission spectra of the probe **Glu-MDA** (10 μM)

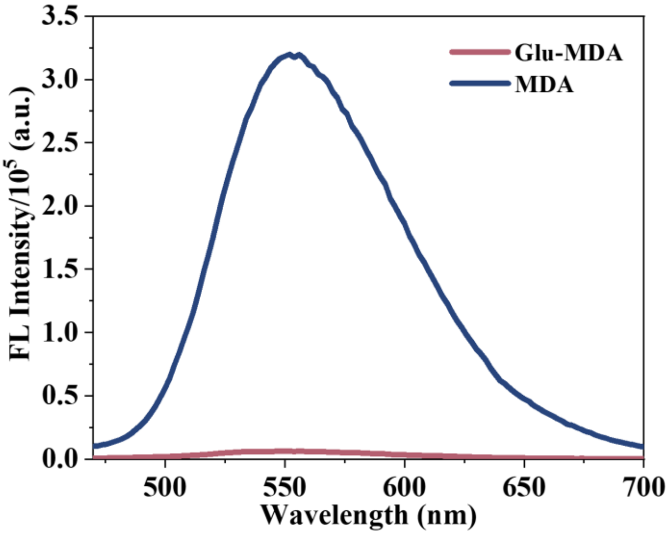

Figure S17. The fluorescence spectra of probe Glu-MDA (10 μM) and fluorophore MDA (10 μM), λ_{ex} = 450 nm

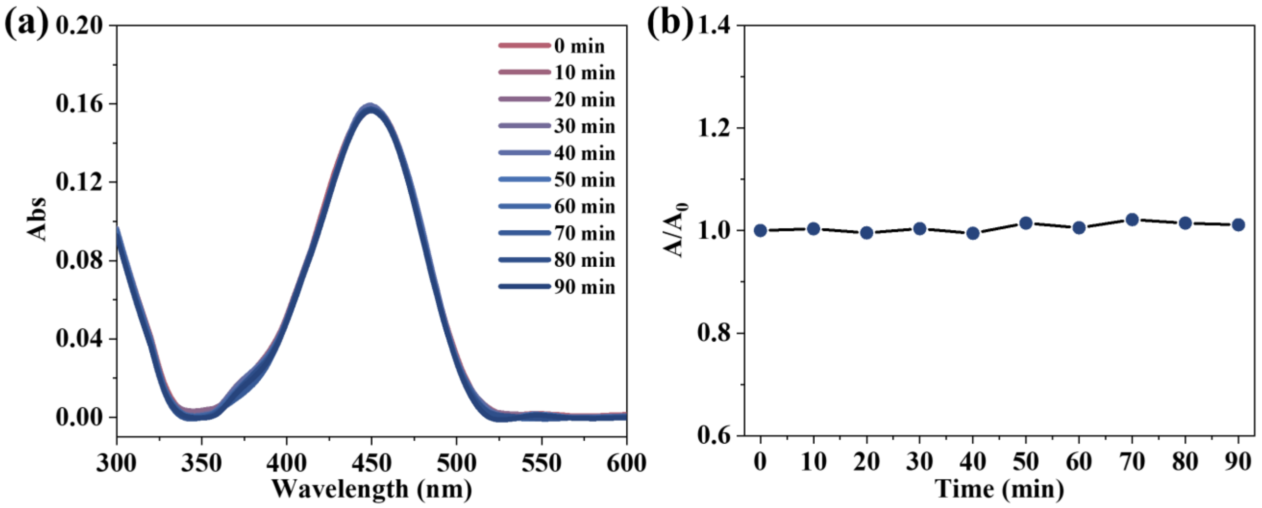

Figure S18. (a) The absorption spectra of the fluorophore **MDA** (10 μM) in DMSO/PBS (1: 4 *v/v*) mixed solution within 90 min and (b) the variation trend with time at 450 nm

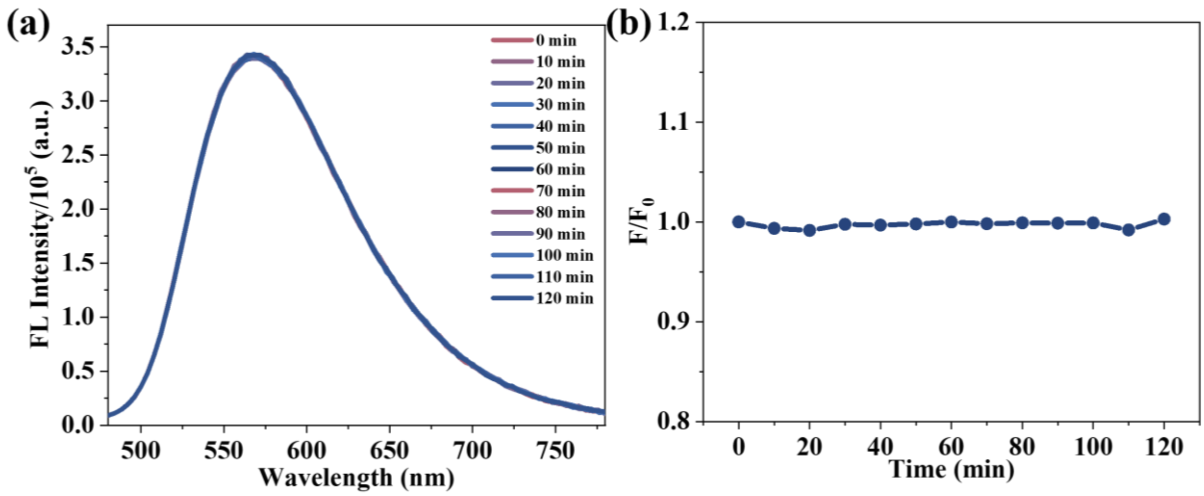

Figure S19. (a) The fluorescence emission spectra of fluorophore **MDA** (10 μM) in DMSO/PBS (1: 4 *v/v*) mixed solution within 120 min and (b) the fluorescence intensity change trend with time at 550 nm

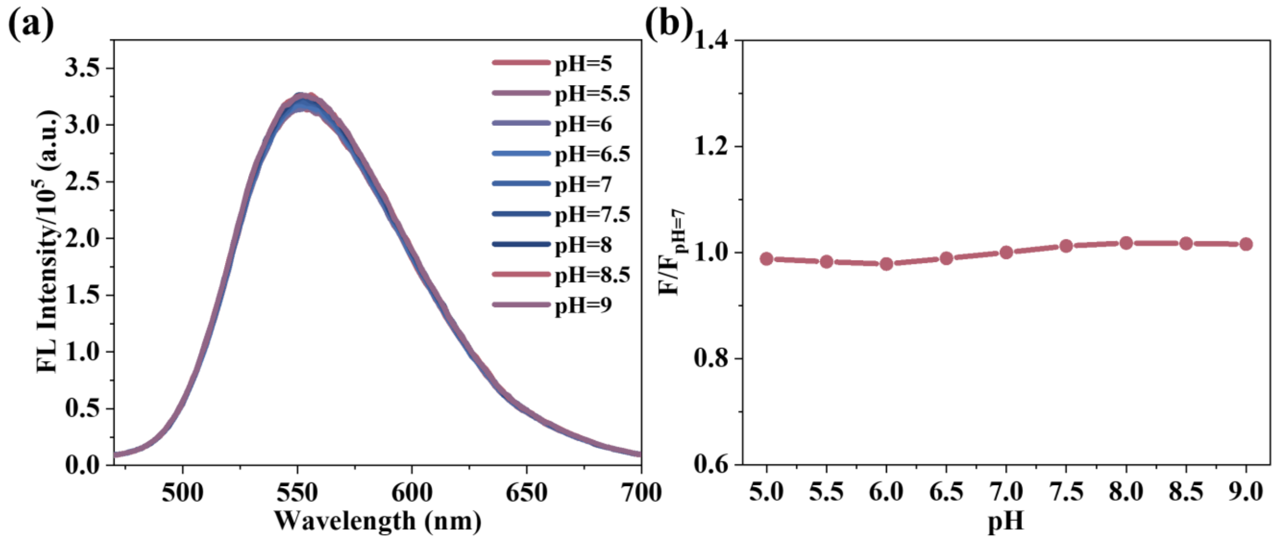

Figure S20. (a) The fluorescence emission spectra of **MDA** (10 μM) in DMSO/PBS (1: 4 *v/v*) mixed solution at different pH and (b) the fluorescence intensity at 550 nm changed with pH

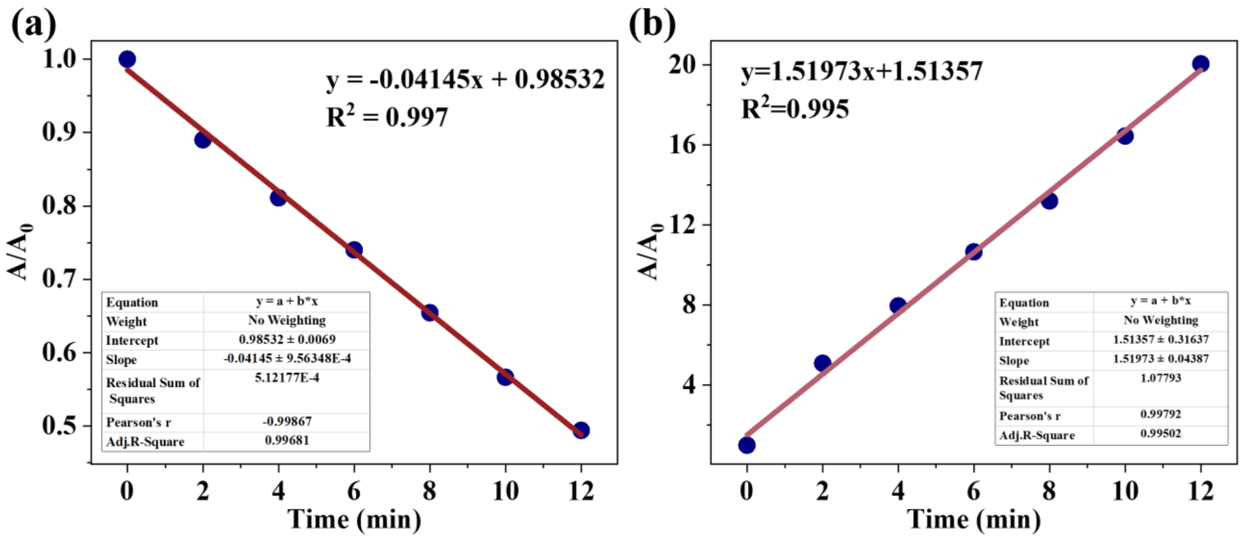

Figure S21. The linear change in absorbance of the probe Glu-MDA (10 μM) over time at (a) 376 nm and (b) 452 nm with the addition of GGT (2 U/L).

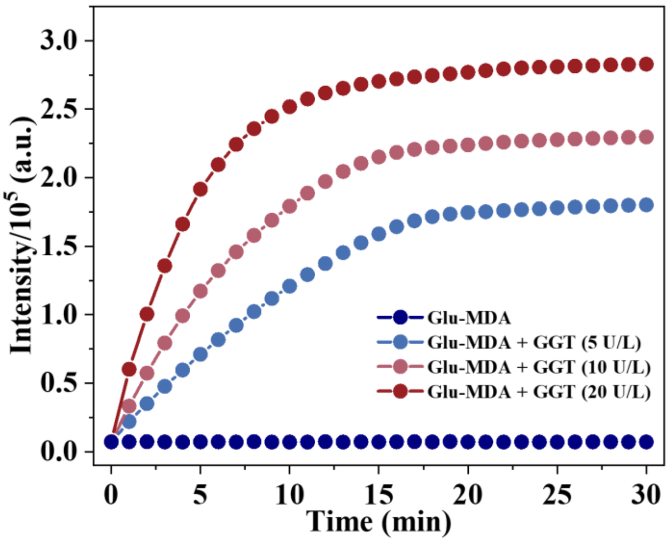

Figure S22. Fluorescence kinetics of **Glu-MDA** (10 μM) on **GGT** (0, 5, 10, and 20 U/L).

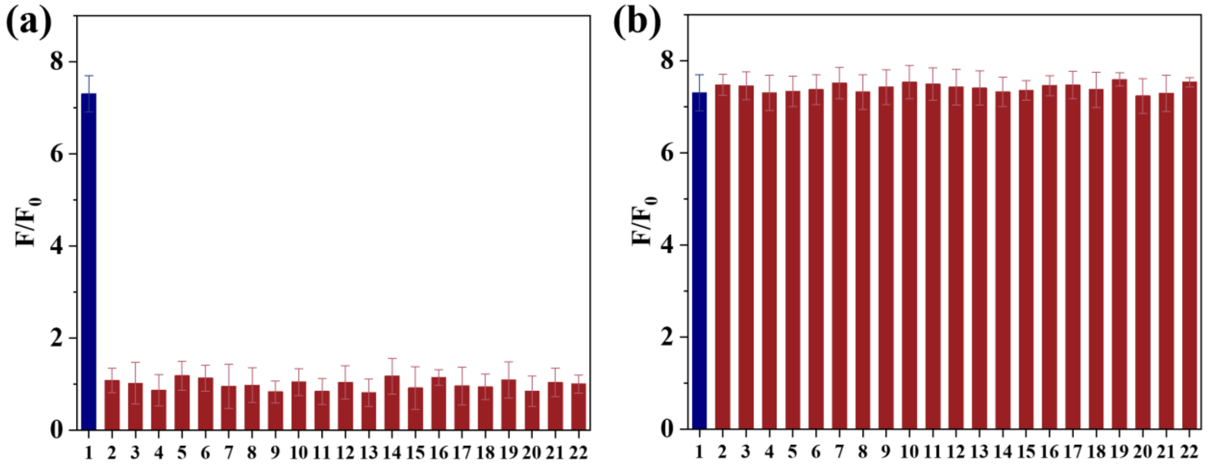

Figure S23. (a) The fluorescence responses of **Glu-MDA** (10 μM) to **GGT** (2 U/L) and various analytes at 550 nm. Analytes 1: 22: **GGT**, $NaNO_3$, $NaNO_2$, Na_2SO_4, Na_2SO_3, NaCl, KCl, $CaCl_2$, H_2O_2, L-ascorbic acid, Glucose, Phe, Pro, Ala, His, Cys, Hcy, GSH, GR, GSH-Px, GST, GCL. Data are presented as the mean value ± standard deviation (SD) (n = 3). (b) Study on the anti-interference of **GGT** detection using **Glu-MDA**. Analytes 1: 22: **GGT**, $NaNO_3$, $NaNO_2$, Na_2SO_4, Na_2SO_3, NaCl, KCl, $CaCl_2$, H_2O_2, L-ascorbic acid, Glucose, Phe, Pro, Ala, His, Cys, Hcy, GSH. GR, GSH-Px, GST, GCL. Data are presented as the mean value ± SD (n = 3).

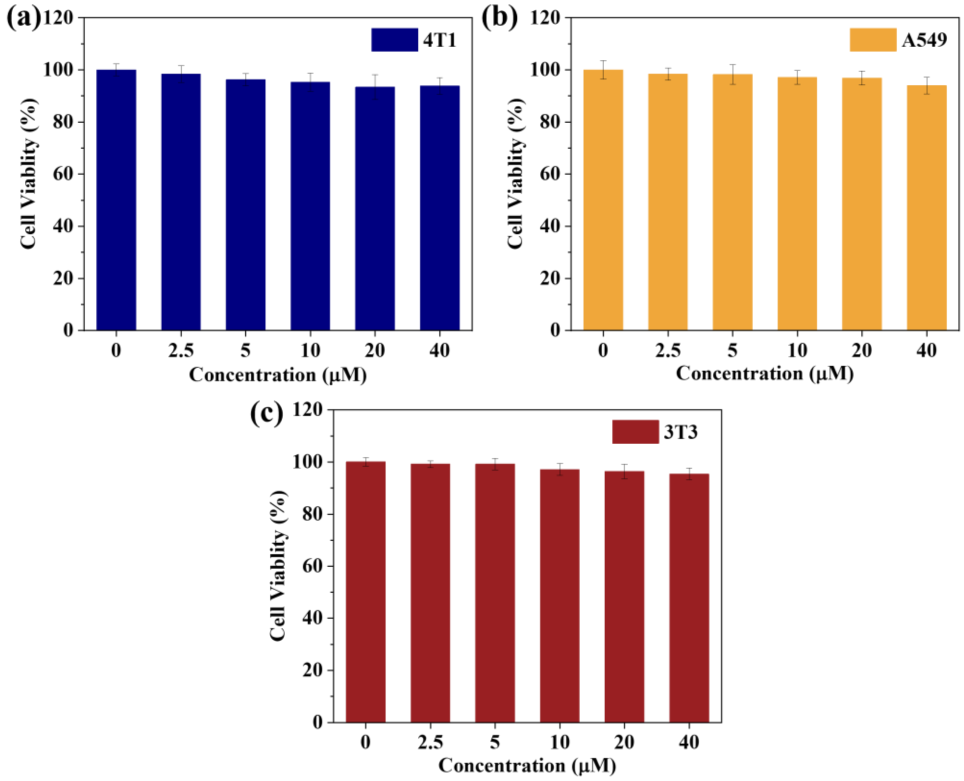

Figure S24. Cytotoxicity of probe Glu-MDA in (a) 4T1 cells, (b) A549 cells, and (c) 3T3 cells cultured in 0-40 μM **Glu-MDA** for 24 h. Data are presented as the mean value ± standard deviation (SD) (n = 3).

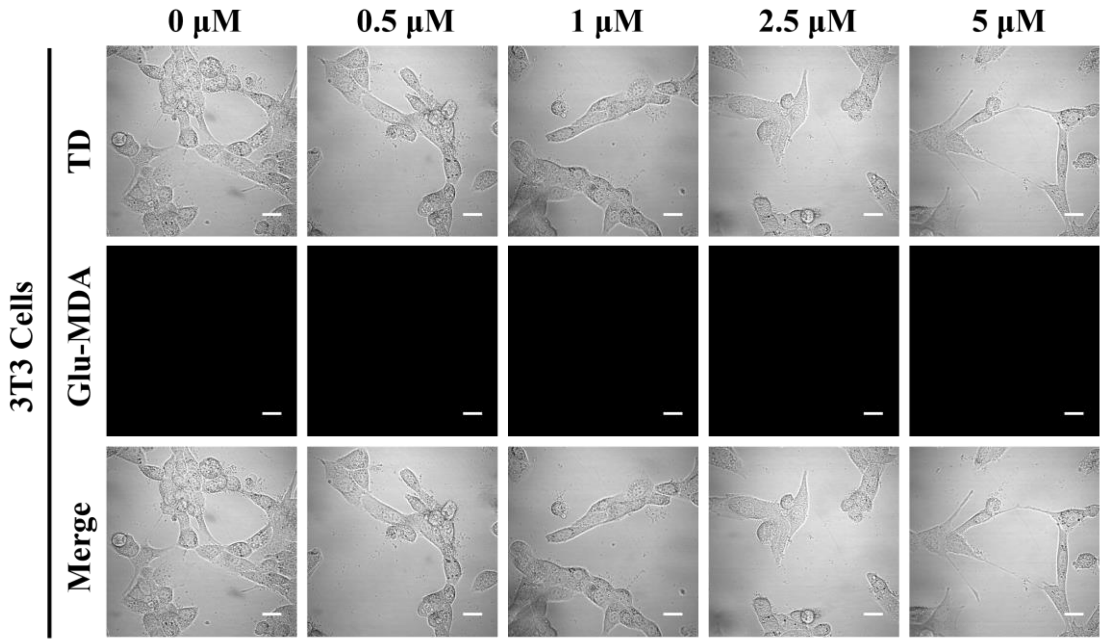

Figure S25. Confocal imaging of endogenous GGT in 3T3 cells labeled with 0, 0.5, 1, 2.5, and 5 μM Glu-MDA. λ_{ex} = 458 nm, red light channel (490-600 nm). Scale bar = 20 μm.

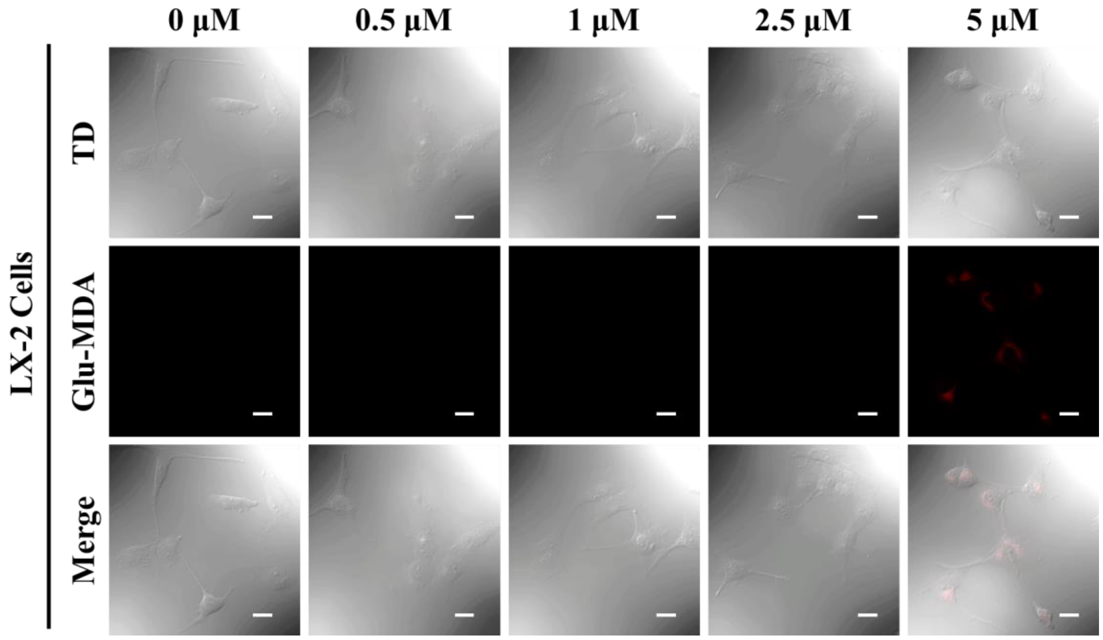

Figure S26. Confocal imaging of endogenous GGT in LX-2 cells labeled with 0, 0.5, 1, 2.5, and 5 μM Glu-MDA. λ_{ex} = 458 nm, red light channel (490-600 nm). Scale bar = 20 μm.

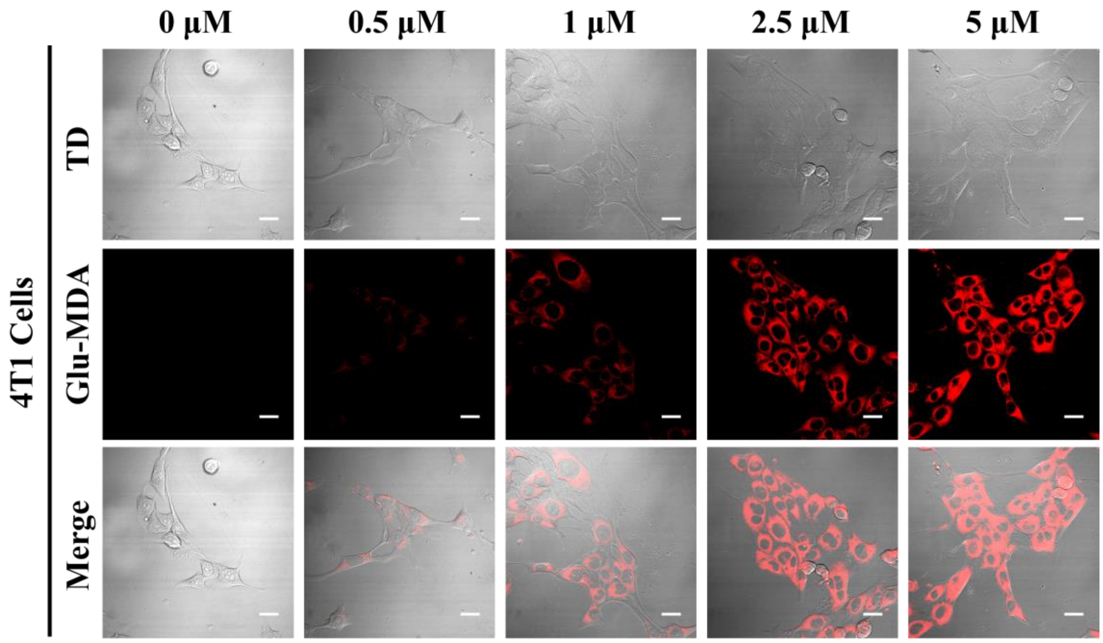

Figure S27. Confocal imaging of endogenous GGT in 4T1 cells labeled with 0, 0.5, 1, 2.5, and 5 μM Glu-MDA. λ_{ex} = 458 nm, red light channel (490-600 nm). Scale bar = 20 μm.

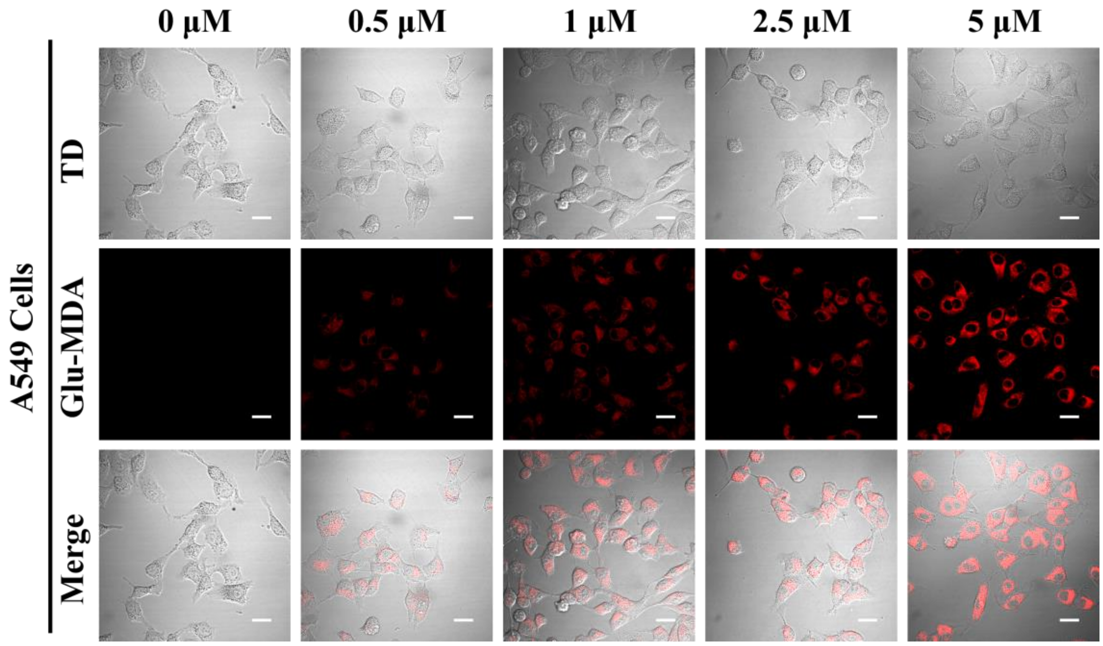

Figure S28. Confocal imaging of endogenous GGT in A549 cells labeled with 0, 0.5, 1, 2.5, and 5 μM Glu-MDA. λ_{ex} = 458 nm, red light channel (490-600 nm). Scale bar = 20 μm.

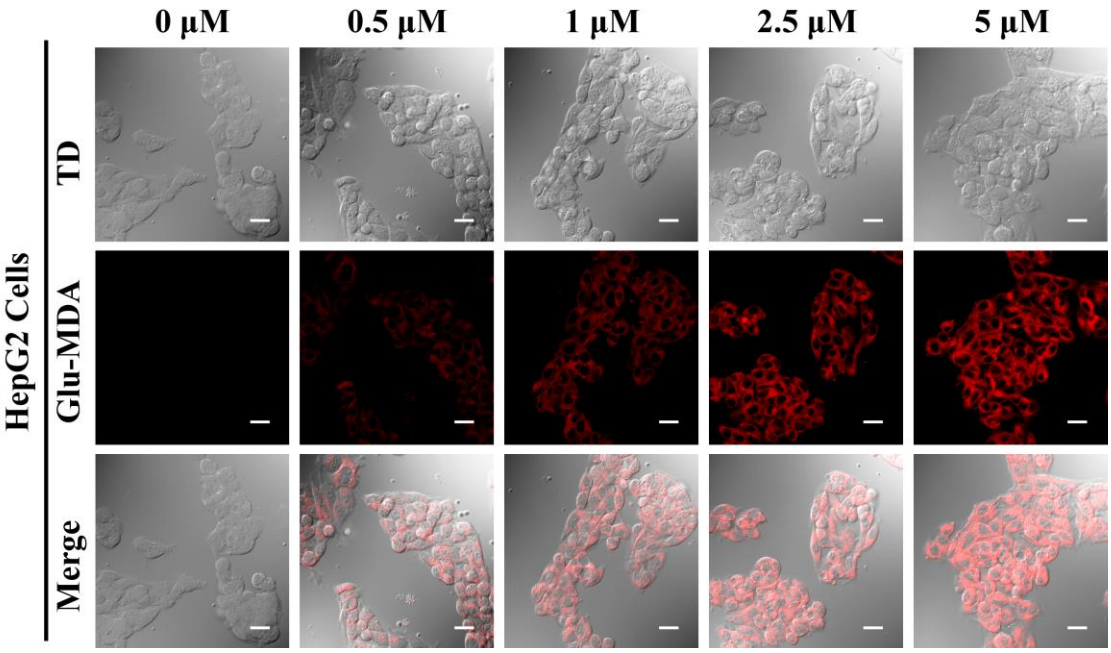

Figure S29. Confocal imaging of endogenous GGT in HepG2 cells labeled with 0, 0.5, 1, 2.5, and 5 μM Glu-MDA. λ_{ex} = 458 nm, red light channel (490-600 nm). Scale bar = 20 μm.

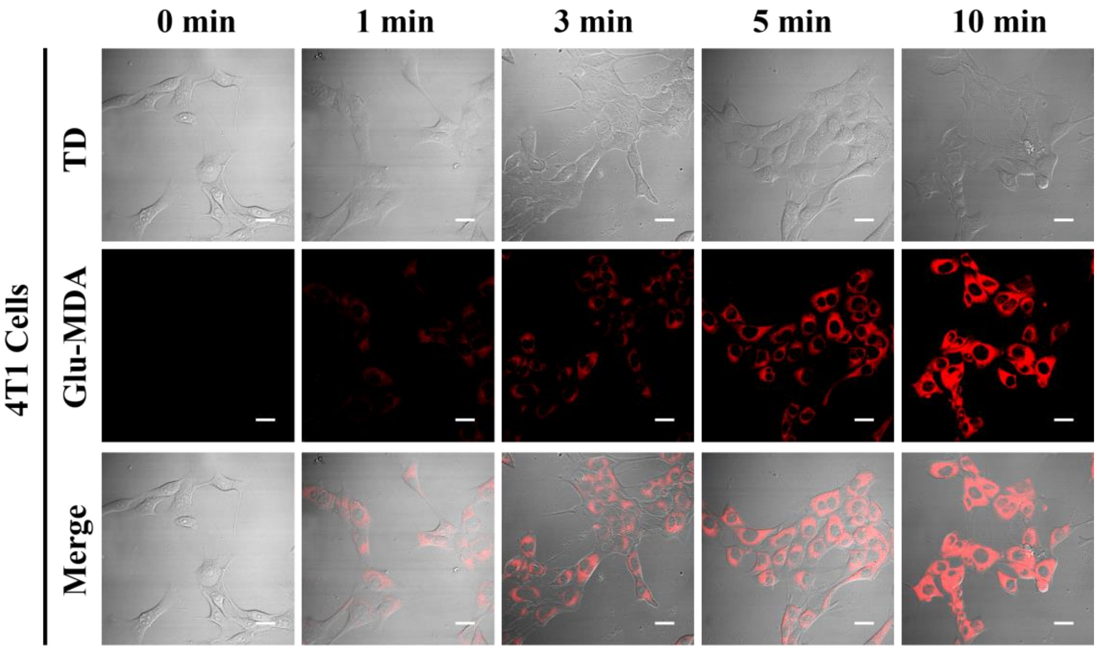

Figure S30. Confocal imaging of 4T1 cells cultured with 2.5 μM Glu-MDA for 0, 1, 3, 5, and 10 min. λ_{ex} = 458 nm, red channel (490-600 nm). Scale bar = 20 μm.

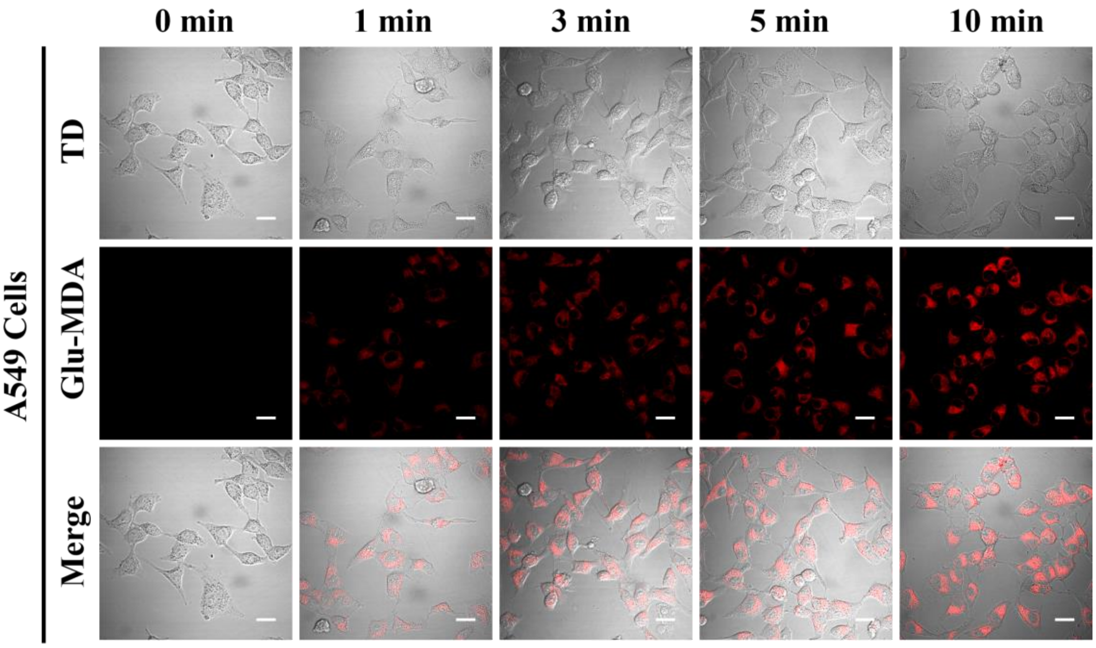

Figure S31. Confocal imaging of A549 cells cultured with 2.5 μM Glu-MDA for 0, 1, 3, 5, and 10 min. λ_{ex} = 458 nm, red channel (490-600 nm). Scale bar = 20 μm.

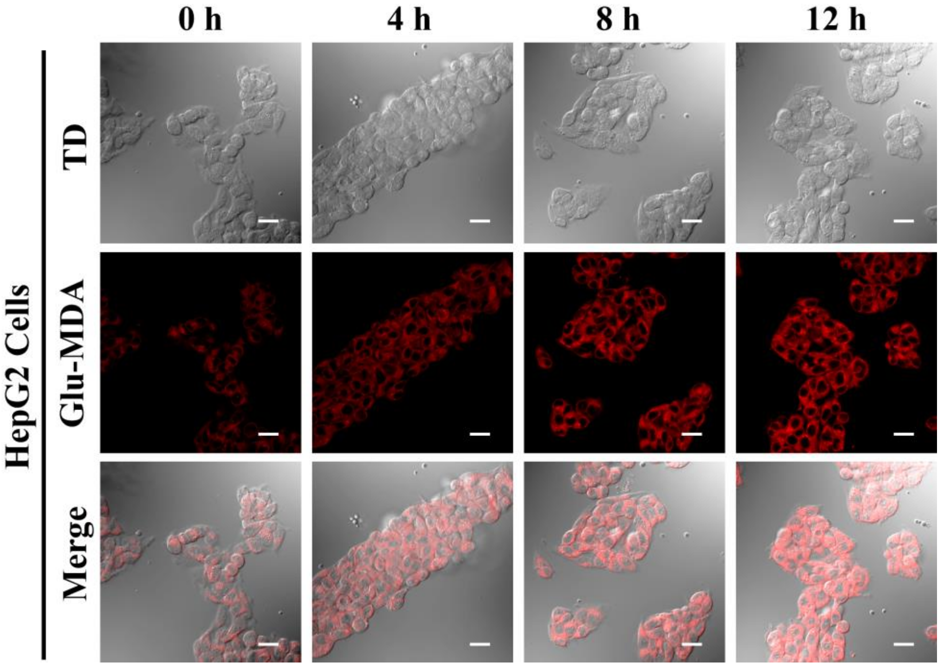

Figure S32. Confocal fluorescence imaging of endogenous **GGT** activity in living HepG2 cells: 2.5 mM sodium butyrate (NaBu)-induced for 0, 4, 8, and 12 h, followed by incubation with 5 μM probe **Glu-MDA** at 37 °C for 10 min. λ_{ex} = 458 nm, red channel (490-600 nm). Scale bar = 20 μm.

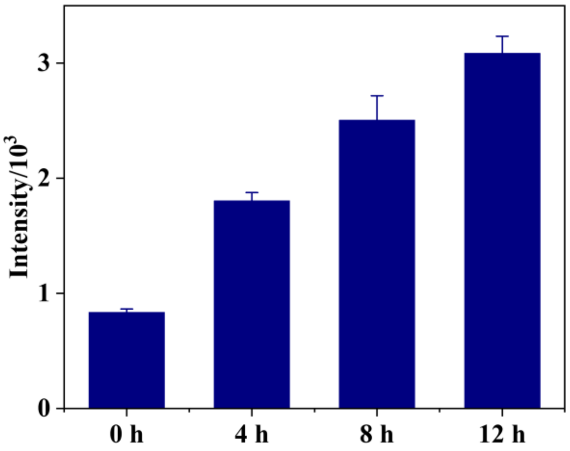

Figure S33. The fluorescence intensity of the **Glu-MDA** probe in HepG2 cells after treating cells with NaBu for 0, 4, 8, and 12 hours, respectively. Data are presented as the mean value ± standard deviation (SD) (n = 3).

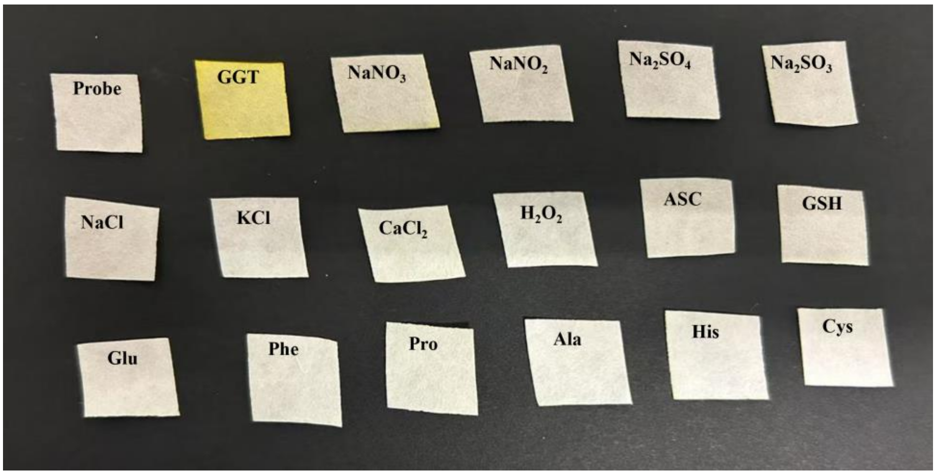

Figure S34. The photograph of the probe **Glu-MDA**-coated filter strips with the different analytes under natural light.

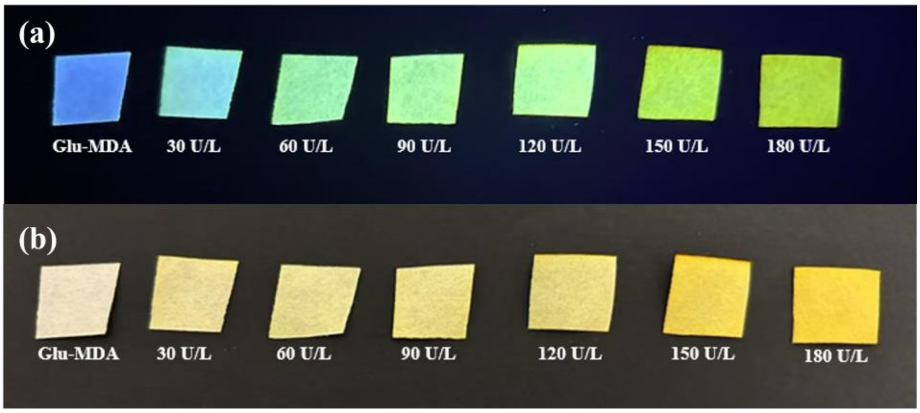

Figure S35. The response of **Glu-MDA** coated filter paper strips to different concentrations of **GGT** (0-180 U/L) under (a) portable UV lamp (365 nm) and (b) natural light.

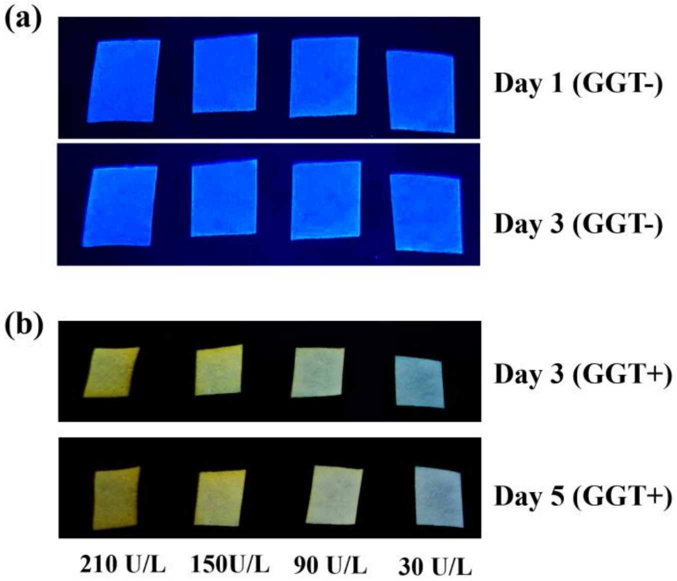

Figure S36. The fluorescence stability of **Glu-MDA** pre-stained test strips (a) before and (b) after detection of different concentrations of **GGT** (210, 150, 90, and 30 U/L).
